# Cancer Burden Attributable to Potentially Modifiable Risk Factors in Australia

**DOI:** 10.3390/cancers17193101

**Published:** 2025-09-23

**Authors:** Tenaw Tiruye, Bereket Duko, Laychiluh Mekonnen, Paul Ward, Trang H. H. D. Nguyen, Stephanie Byrne, David Roder, Kerri Beckmann

**Affiliations:** 1 Cancer Epidemiology and Population Health Research Group, Allied Health and Human Performance, University of South Australia, Adelaide 5000, Australia; 2 School of Public Health, Debre Markos University, Debre Markos P.O. Box 269, Ethiopia; 3 Research Centre for Public Health, Equity and Human Flourishing (PHEHF), Torrens University Australia, Wakefield Street Campus, Adelaide 5000, Australia; 4 Curtin School of Population Health, Curtin University, Bently 6102, Australia; 5 Clinical and Health Sciences, University of South Australia, Adelaide 5000, Australia; 6 School of Public Health, University of Adelaide, Adelaide 5005, Australia; 7 Allied Health and Human Performance, University of South Australia, Adelaide 5000, Australia

**Keywords:** neoplasms, global burden of disease, disability-adjusted life years, death, Australia

## Abstract

Understanding the relative contribution of modifiable risk factors to cancer morbidity and mortality is crucial for designing effective cancer prevention and control strategies. Our study estimated cancer-related deaths and disability-adjusted life years (DALYs) lost attributable to potentially modifiable risk factors in Australia using data from the Global Burden of Diseases 2021 study. In 2021, an estimated 20,409 cancer deaths (37.5%) and 431,575 cancer DALYs lost (37.9%) in Australia were attributable to potentially modifiable risk factors. Males had higher modifiable risk attributed to cancer death and DALY rates than females. Behavioral risks accounted for 25.0% of cancer deaths and 26.5% of DALYs. Metabolic risks and environmental/occupational risks accounted for 9.4% and 9.3% of deaths, respectively. Smoking remained the leading attributable risk factor, accounting for 12.2% cancer deaths and 13.1% DALYs lost. Dietary risks accounted for 40.0% of colorectal cancer deaths and DALYs lost. Cervical, larynx, liver, lung, and colorectal cancers had a high proportion of deaths and DALYs lost attributed to modifiable risks. Liver and nasopharyngeal cancers had the highest burden attributed to alcohol use (39.1% and 39.0%, respectively), while 21.3% liver cancer deaths were attributed to drug use. Strengthening public health interventions, such as multi-disciplinary approaches to promote a healthy lifestyle, is required.

## Simple Summary

This study provides an up-to-date estimate of Australia’s cancer burden attributable to modifiable risk factors. The results highlighted that one third of cancer deaths and disability-adjusted life years (DALYs) lost are preventable through addressing common risk factors. This research is timely, comprehensive in its scope of risk factors and cancer types, uses DALYs for a holistic cancer burden assessment, and includes detailed sex-specific analysis. The results provide crucial evidence for targeted public health measures that reduce preventable risks.

## 1. Introduction

While advancements in diagnosis and treatment have improved cancer outcomes, the overall burden of cancer continues to grow [1]. A substantial portion of this burden is attributable to modifiable risk factors that encompass a wide range of lifestyle factors and environmental exposures [1,2,3]. Targeting these modifiable risk factors is likely to provide one of the most effective and cost-efficient strategies for reducing both cancer incidence and mortality [4]. Understanding the magnitude of these preventable cancer burdens is therefore crucial for informing public health strategies and reducing the impact of cancer on population health.

An extensive body of research from Australia has explored the contribution of various risk factors to cancer incidence and mortality [5,6,7,8,9,10,11,12,13,14,15]. However, this literature has largely focused on a limited number of cancer types and specific risk factors, predominantly smoking, alcohol, and overweight/obesity [5,6,7,9,10,11,12,14,16]. Few studies have investigated the contribution of modifiable risk factors to the non-fatal burden of cancer (disability) [6,17]. Overall, recent detailed evidence within the Australian/local context is lacking—this is particularly important given that attributable risks vary significantly by setting (e.g., sociocultural, environmental, economic, and health system responses). The Global Burden of Diseases, Injuries, and Risk Factors Study (GBD) framework provides a unique opportunity for analysis of the proportion of cancer deaths and cancer-related burden attributable to a wide range of risk factors for a more comprehensive list of cancer types. Yet, no recent GBD studies have explored this for the Australian population. While recent GBD studies have examined disease burden and risk factors globally or within Australia, they often lack sufficient detail. Some analyses are limited by a lack of cancer-specific information [18,19], a focus on specific age groups [18], or do not disaggregate results at the national level for Australia [1,20]. This highlights the need for a more comprehensive and nationally tailored analysis of modifiable cancer risk factors in Australia. The most recent comprehensive study, using GBD2015 data [17], assessed the broader impact of behavioral/lifestyle risks and environmental/occupational exposures on the overall cancer burden in Australia. Given shifts in population risk profiles, including the potential impacts of screening, diagnoses, cancer prevention strategies, and cancer services, updated analyses using more recent GBD data are warranted. Furthermore, the GBD enables a direct comparison of risk–cancer pairs within a consistent global framework and provides standardized attributable fractions that may not be routinely available from national cancer registries. The GBD study’s consistent methodology and regular updates also enable longitudinal analysis over a period stretching back to 1990. While the Australian Burden of Disease studies, which are produced by the Australian Institute of Health and Welfare (AIHW), provide disease burden estimates, they lack global comparability and cover a wide set of risk–cancer pairs.

This study aims to provide a comprehensive and up-to-date assessment of preventable cancer deaths and DALYs lost in the Australian context by quantifying the cancer burden attributable to potentially modifiable risk factors, using data and methodology of the GBD2021 study. The GBD2021 study provides global cancer burden estimates, including incidence, mortality, disability-adjusted life years (DALYs) lost, and risk factor attributions. We emphasized risk attributable to cancer deaths and DALYs lost, the latter often overlooked in the available evidence. DALYs provide a comprehensive measure of the overall disease burden (both fatal and non-fatal burden), combining years of life lost due to premature mortality and years lived with disability [21].

## 2. Materials and Methods

The GBD2021 study employed a systematic approach to quantify health loss from major diseases, injuries, and risk factors across populations worldwide. The study leveraged a wide array of data sources, including disease registries, vital registration systems, household surveys, hospital records, clinical data, reports, and scientific literature. These data were collated and analyzed using standardized methods. Subsequently, these data underwent rigorous quality control and further standardization processes to ensure their accuracy and comparability. The GBD employed Bayesian meta-regression disease modeling (DisMod-MR 2.1) and causes of death ensemble modeling (CODEm) methods to calculate fatal and non-fatal health metrics over time [22].

The GBD2021 study includes estimates for the global burden of disease due to cancers, including both common and rare malignancies, allowing exploration of the impact of various exposures on cancer burden. Within the GBD database, the relationship between each risk factor and health outcomes can be analyzed to estimate the relative risk of each risk factor on the outcome (e.g., incidence, deaths, or DALYs). The population attributable fraction (PAF), which accounts for the competing risk of death, risk factor interdependence, and statistical uncertainty, is then used to predict the proportion of health risk that could be reduced if exposure to the risk factor is lowered to the minimum level. A detailed description of the GBD study aims, methodology, data sources, and analytic tools has been reported previously [23].

To handle the non-independent, synergistic effects of multiple risk factors (e.g., how diet and obesity together increase cancer burden more than the sum of their individual effects), GBD uses a hierarchical, counterfactual approach [23]. This method attributes the burden sequentially, giving priority to risks that are more causally “upstream,” such as metabolic risks over behavioral ones, and it uses a joint exposure distribution to avoid double-counting the burden. This allows GBD to produce internally consistent estimates of the total attributable burden across all risk factors, ensuring that the total burden attributed to all risks does not exceed 100% of the overall disease burden [23].

For our study on cancer burden attributable to risk factors in Australia in 2021, results were extracted and compiled from the GBD Compare (https://vizhub.healthdata.org/gbd-compare/cancer) and Global Health Exchange websites (https://ghdx.healthdata.org/) accessed on 13 January 2025. Our outcomes of interest were cancer deaths and DALYs lost. One DALY represents one year of “healthy life” lost due to illness/disability and/or premature death [21]. From the GBD database, we extracted data for causes identified as “neoplasms”, measurements of “death” and “DALY”, and location specified as “Australia”, with metrics including “number”, “percent”, and “rate”. “Risk factors” included all GBD risk factors (explored at both level 1 to understand the role of the three major GBD risk classifications, and levels 2 and 3 for detailed insights into specific risks). The three GBD level-1 risk categories are behavioral, metabolic, and environmental/occupational risks. Behavioral risks include tobacco (smoking, second-hand smoke and chewing tobacco), dietary risks (diet high in processed meat, diet high in red meat, diet high in sodium, diet low in calcium, diet low in fiber, diet low in fruit, diet low in milk, diet low in vegetables, diet low in whole grains), high alcohol use, drug use, physical inactivity, and unsafe sex. Metabolic risks include high body mass index (BMI) and high fasting plasma glucose. Environmental/occupational risks include occupational carcinogens, particulate matter pollution, and residential radon. Overall, 23 specific cancer types (and all cancers combined) and 27 risks (all risks combined, 3 GBD level-1 risks, 8 GBD level-2 risks, and 15 GBD level-3 risks) were included (Table 1).

A total of 159 risk–cancer combinations were estimated. The list of risk factors at the GBD hierarchy (levels 1, 2, and 3) and risk–cancer combinations is described in Table 2.

The attributable risk effect measure was estimated as the proportion of cancer deaths and DALYs that can be attributed to a specific risk factor (e.g., DALYs lost due to cancer attributable to behavioral risk factors). The risk-attributed metrics were computed along with a 95% Uncertainty Interval (UI, calculated as the 2.5th and 97.5th percentile values). The results were summarized as estimated numbers, attributed percentages, and age-standardized rates per million population, and presented by sex (male, female, and both).

## 3. Results

### 3.1. Cancer-Related Deaths and DALYs Lost in Australia

In 2021, the estimated total number of cancer deaths in Australia was 54,428 (95% UI: 49,281–57,750). Cancer contributed to almost a third of all deaths in Australia (31.1%) in the same year, with a higher proportion of deaths among males than females (33.8% vs. 27.9%). Cancer DALYs lost overall were estimated to be 1,139,729 years (95% UI: 1,057,124–1,195,026), with males experiencing higher DALYs lost than females (652,347 vs. 487,381 years). Lung cancer was the leading cause of death in both sexes, with a significantly higher proportion in males (6.6%) compared to females (5.0%). Prostate cancer in males (4.8%) and breast cancer in females (4.3%) were the second leading causes of cancer deaths in each sex. Colorectal, liver, and pancreatic cancers also contributed significantly to both deaths and the loss of healthy life years across both sexes (Table 3).

### 3.2. Risk-Attributable Cancer Deaths in Australia

**Figure 1:**
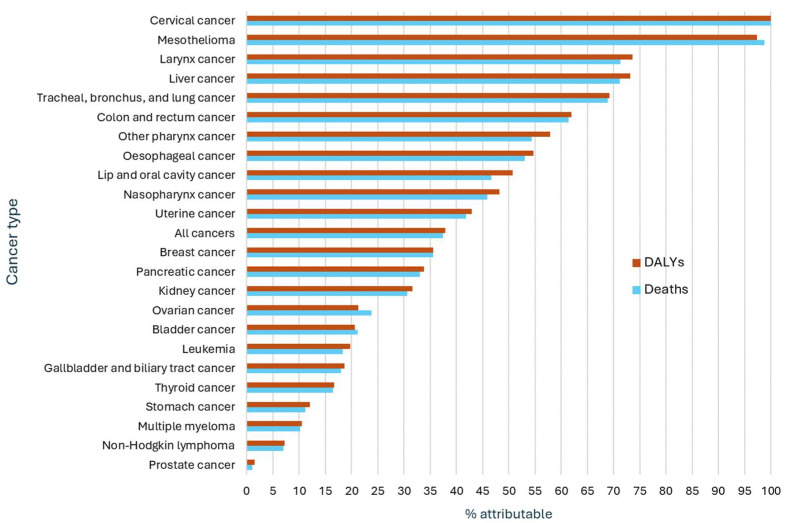
Cancer-related deaths and DALYs lost attributed to all GBD risk factors in Australia in 2021, both sexes.

**Figure 2:**
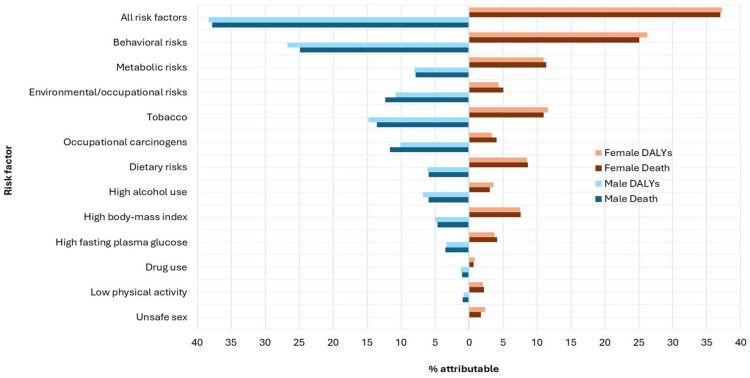
All cancer deaths and DALYs lost attributed to selected GBD risk factors in Australia in 2021.

In Australia in 2021, an estimated 20,409 (37.5%) cancer deaths (37.8% in males and 37.0% in females) were attributable to potentially modifiable risk factors. Males had higher risk-attributable cancer death rates compared to females (548.7/million vs. 342.7/million people). Behavioral risks combined (tobacco, dietary risks, high alcohol use, drug use, physical inactivity, and unsafe sex) contributed to 13,600 (25%) cancer deaths, corresponding to 298.4 deaths per million population. Behavioral risks made the greatest contributions to deaths from cancer of the cervix (100%), larynx (63.7%), liver (63.0%), other pharynx (oropharynx and hypopharynx, excluding nasopharynx) (54.4%), esophagus (53.0%), and colorectum (51.6%). Metabolic risks and environmental/occupational risks accounted for 9.4% and 9.3% of cancer deaths (108.2 and 103.1 deaths per million people, respectively). Metabolic risk factors contributed most to deaths from pancreatic (27.0%), kidney (25.8%), liver (21.4%), and colorectal (20.7%) cancers, while environmental/occupational risk factors contributed greatly to mesothelioma (98.8%) and lung cancer (39.1%). Cancer deaths attributed to environmental/occupational risks were higher in males than females (12.3% vs. 5.1%), whereas deaths attributed to metabolic risks were higher in females than males (7.9% vs. 11.4%) (Table 4, Figure 1 and Figure 2).

Almost all mesothelioma and cervical cancer deaths were attributed to modifiable risks. Nearly 100% of cervical cancer deaths were attributable to unsafe sex. Across both sexes, larynx (71.3%), liver (71.2%), lung (68.9%), and colorectal (61.5%) cancers also exhibited high-risk-attributable deaths. Risk-attributable death rates were higher in males than females for cancers of the larynx, lip and oral cavity, liver, nasopharyngeal, esophagus, other pharynx, and lung. Stomach cancer, multiple myeloma, and non-Hodgkin lymphoma had the lowest proportions of risk factor attributable deaths across both sexes (11.2%, 10.2% and 7.0%, respectively). Only 1.0% of prostate cancer deaths were attributable to modifiable risk factors (Table 4, Figure 1 and Figure 2).

Regarding specific risk factors, smoking was the largest contributor to all cancer deaths in both sexes (13.3% in males and 10.8% in females). Approximately 4511 lung cancer deaths in 2021 were attributable to smoking alone, accounting for 44.7% of lung cancer deaths in males. Dietary risks combined accounted for about 39.5% of colorectal cancer deaths in both sexes. Diet low in vegetables accounted for 21.0% esophageal cancer deaths, and a diet low in whole grains accounted for 17.0% of colorectal cancer deaths. Drug use is a highly attributable risk for liver cancer deaths (21.3%). Alcohol use is attributed to 6.0% of all cancer deaths in males, mainly for liver, lip, and oral cavity, and nasopharyngeal cancers. High BMI contributed to 7.6% of all cancer deaths in females, primarily towards uterine and kidney cancers (Table 4, Figure 1 and Figure 2).

### 3.3. Risk-Attributable Cancer DALYs Lost in Australia

Approximately 431,575 (37.9%) of DALYs lost due to cancer in Australia in 2021 (38.3% in males and 37.2% in females) were attributable to potentially modifiable risk factors. The pattern for cancer DALYs lost attributable to risk factors was similar to patterns observed for risk-attributable cancer deaths. Behavioral, metabolic, and environmental/occupational risks accounted for 302,471 (26.5%), 105,916 (9.3%), and 91,965 (8.1%) of all cancer DALYs lost, respectively. DALYs lost from cervical cancer due to unsafe sex (100%) and from mesothelioma due to occupational carcinogens (97.4%) topped the list. Larynx, liver, lung, and colorectal cancers also had high proportions of risk-attributed DALYs lost (73.6%, 73.2%, 69.2%, and 62.0%, respectively) (Table 5, Figure 1 and Figure 2).

Similar to risk-attributable cancer deaths, smoking stands out as the highest contributor to cancer DALYs lost, accounting for 149,615 (13.1%) DALYs lost in Australia in 2021 across both sexes, with a greater contribution in males than females. Smoking-attributable DALYs lost were also high for cancers of the larynx (58.4%), lung (47.9%), other pharynx (29.0%), and esophagus (22.6%). Other notable behavioral risks contributing to high DALYs lost included dietary risks and alcohol use, accounting for 7.1% and 5.4% of all cancer DALYs, respectively. Dietary risks accounted for 39.7% of colorectal cancer DALYs lost, with a diet high in red meat contributing 17.2% and a diet low in whole grains contributing 17.8%. A diet low in vegetables was a significant contributor to DALYs lost in esophageal cancer (20.8%). Alcohol use accounted for 41.6%, 41.2%, and 40.3% of DALYs lost due to nasopharyngeal, other pharynx, and liver cancers, respectively. Occupational carcinogens and high BMI contributed to 82,345 (7.2%) and 69,055 (6.1%) all cancer DALYs lost, respectively. Other notable risk—attributable impacts on DALYs lost included occupational carcinogens with lung cancer (30.7%), high BMI with kidney cancer (26.5%), high fasting plasma glucose with pancreatic cancer (23.6%), drug use with liver cancer (22.4%), and a diet low in vegetables with esophageal cancer (20.8%) (Table 5, Figure 1 and Figure 2).

## 4. Discussion

This study provides an up-to-date examination of cancer burden in Australia attributable to potentially modifiable risk factors. The results highlighted that a significant portion of cancer burden in Australia is preventable through addressing common modifiable risk factors. The novelty of this study lies in its timeliness (using the latest GBD 2021 data), its comprehensiveness (in terms of risk factors and cancer types), its emphasis on DALYs lost for a holistic cancer burden assessment, and its detailed, sex-specific analysis that directly addresses identified limitations in the existing Australian literature. It provides critical, current evidence on the preventable portion of cancer deaths and DALYs, which can inform targeted public health measures and lead to significant public health gains at a population level.

In 2021, cancer was responsible for 31% of all deaths and 17% of healthy life years lost in Australia. Notably, 38% of all cancer deaths and cancer-related DALYs lost were attributable to potentially modifiable risk factors. The results are in line with the 2024 Australian Burden of Disease study, showing 39.9% DALYs of cancer were due to modifiable risk factors [24]. While the Australian Burden of Disease study [24] provides a means to interactively explore broader disease burdens and the risk factors with more recent and ongoing analysis of trends, our study provides comprehensive details of how these risk factors contribute specifically to cancer-related deaths and DALYs lost.

Behavioral risk factors continue to play a critical role in driving the cancer burden in Australia. Our study findings indicate that behavioral risk factors (including tobacco and alcohol use, as well as dietary habits) were major contributors to cancer-related deaths and DALYs lost. Cervical cancer-related deaths and DALYs lost were almost entirely associated with preventable risks: unsafe sexual practices (HPV infection). While Australia’s HPV vaccination program is widely considered successful in reducing cervical cancer, challenges persist in achieving higher vaccine coverage. Key barriers include absenteeism, issues with consent form return, and general vaccine hesitancy [25]. Furthermore, significant disparities in vaccine uptake exist among Indigenous populations due to social, cultural, information, and communication-related barriers [26]. Therefore, implementing measures to enhance the program’s reach and address these specific barriers is crucial for maximizing its impact.

Furthermore, dietary habits and low physical activity significantly contributed to colorectal cancer deaths and DALYs lost. These findings align with results of previous studies, both globally and specific to Australia [1,3,19]. This reflects broader global health patterns, where lifestyle factors are increasingly recognized as key contributors to non-communicable diseases, including cancer [1]. This underscores the considerable potential for cancer prevention through effective strategies that address these risks, such as multi-disciplinary interventions to promote healthy lifestyles and “fat” tax policies [27]. It is also important to view lifestyle and behavioral risks through the lens of the profound influence social, economic, and commercial factors have on shaping health behaviors. For instance, limited access to affordable and healthy food options and the absence of safe and accessible spaces for physical activity are not merely individual choices but rather consequences of broader structural inequities [28]. This implies that targeting only the “behavior” without addressing its social and economic determinants could make it difficult to reduce the risk factors.

Tobacco control measures implemented in Australia over recent decades [29] have resulted in a decline in the smoking-attributable cancer burden [30,31]. The success of these tobacco control measures provides valuable lessons for scaling up and transferring successful public health strategies towards tackling other modifiable risk factors. However, our study and a previous study [32] demonstrated that smoking continues to be the leading single modifiable risk factor for lung cancer and the overall cancer burden. This could reflect the consequences of high smoking rates in the mid-late 1900s to early 2000s, due to relatively long lead times for smoking-related cancers. Furthermore, the reduction in smoking-attributable cancer burden may not be uniform across different population subgroups. For example, tobacco use accounted for 37% of the cancer burden for Indigenous Australians in 2018, while it accounted for 9.2% of the cancer burden in the general population [33]. Another study showed that although smoking-attributable lung cancer has reduced over time, high underlying risk estimates were observed among disadvantaged, remote, and Indigenous female Queenslanders [34]. Smoking rates have also not declined as much in socioeconomically disadvantaged groups as they have in the general population [35], and vaping and tobacco pouches are increasing among younger people [36]. Further targeted smoking cessation strategies are needed specifically targeting these population groups.

Our study shows that excessive alcohol consumption is linked to a high proportion of liver and esophageal cancer deaths and DALYs, while drug use is considerably linked with liver cancer burden. A study conducted in New South Wales, Australia, revealed that for each additional seven alcoholic beverages consumed weekly, the relative risk of mortality from alcohol-related cancers increases by 12%, accounting for 3.4% of all cancer fatalities [37]. These findings underscore the importance of strengthening public health measures aimed at reducing alcohol and drug use. Efforts such as public awareness campaigns, pricing and restricting promotions, and warning labels on alcohol products highlighting the link between alcohol and cancer are needed.

Our analysis suggested that adopting behavior modifications such as a healthy diet, smoking cessation, reduced alcohol consumption, and increased physical activity could have prevented 52% of colorectal cancer deaths and lost DALYs. The importance of interventions targeting multiple risk factors in combination has been previously suggested. A study from Australia highlighted the value of managing multiple risk factors to improve cancer survival in primary care for older individuals, rather than considering individual risk factors in isolation [38]. Promoting physical activity as a component of cancer prevention programs in Australia is also crucial, a finding supported by our study and previous research [8,39,40] showing that physical inactivity contributes to cancer burden. Furthermore, some risk reduction interventions can have a synergistic effect on other cancer prevention efforts, such as screening. For example, a cross-sectional study of 1685 Australian women aged 44–48 and 64–68 years demonstrated that effectively targeting multiple risk factors (current smoking, not working, obesity, anxiety, poor physical health, no children, no partner, receiving income support payments, and low overall health service use) could potentially reduce overall non-participation in cervical cancer screening by 74% [41].

In line with a previous global estimate of cancer burden using GBD2019 data [1], our study indicated that metabolic risk factors (high BMI and high fasting plasma glucose) have seen the largest increases in cancer burden, while behavioral and environmental/occupational risks declined between 2010 and 2021 in Australia (results not reported). Our study also highlighted the strong link between high fasting plasma glucose and pancreatic cancer. Another study showed that high BMI attributed to cancer mortality has increased in the Asia-Pacific region [42]. These trends emphasize the need for continuous monitoring of cancer trends and their association with specific risk factors to inform more targeted prevention efforts. This includes monitoring the potential need to shift investment towards risk reduction and prevention strategies and developing public health campaigns to address these changing risk profiles. In addition to behavioral interventions, addressing obesogenic environments through urban planning (e.g., controlling fast food density), restricting the marketing of junk food in all forms of media (especially to children to reduce patterned behaviors), and providing subsidies for healthy food to low-income families could help.

Our results highlighted notable sex-specific differences in the cancer deaths and DALYs lost attributed to risk factors, with the overall burden being greater for males. Some risk factors also affected one sex more significantly than the other and varied by cancer site. For example, the smoking- and alcohol-attributable burden of lung, liver, and laryngeal cancers was higher in males, while high BMI contributed more to the cancer burden in females. Previous evidence showed that the burden of smoking [7,9,10,16] and alcohol consumption is often higher in men [9], while high BMI contributes to cancer burden slightly more in women [6,10], especially in postmenopausal women [5]. Although some of these disparities may be related to physiological and/or lifestyle differences [43,44], the findings underscore the need to examine cancer control efforts through a gendered lens. Prevention strategies targeting men that challenge social norms have the potential to reduce cancer burden [45].

Our findings show that a substantial portion of the cancer burden (62% of deaths and DALYs lost) was not attributed to any of the GBD risk factors examined, suggesting they may be unavoidable or risk factors that are yet unknown. This implies that merely preventing the reported risk factors would not eliminate all cancer deaths and/or DALYs lost. Instead, a combination of interventions across the cancer control continuum is required, including early detection and timely diagnosis, as well as quality cancer treatment and survivorship care. It is also worth noting that the contribution of modifiable risk factors for some cancers, such as prostate cancer, is small and cannot be controlled merely with primary prevention measures. The Lancet Commission on Prostate Cancer indicates that the rise in prostate cancer cases cannot be entirely mitigated through lifestyle modifications or public health initiatives, as there is a significant genetic risk of prostate cancer [46]. This evidence underscores the importance of considering family history and genetic testing in these cohorts of patients. Public health initiatives promoting screening among high-risk groups, such as providing information and tools for people over 50 to make informed decisions about screening, would also reduce cancer deaths and DALYs.

Finally, social determinants of health, such as socioeconomic disadvantage, education, housing, employment, and access to nutritious food and healthcare, may create the conditions in which health decisions are made, with economically disadvantaged communities often facing limited access to health-promoting resources [28,47,48]. Moreover, structural and commercial determinants—including urban planning, marketing practices by tobacco and alcohol industries, and inequities in healthcare access—create environments of elevated risk for marginalized populations [49]. Equity-focused public health policies and cross-sectoral collaboration are essential for reducing cancer disparities and shifting the focus from individual behavior to systemic drivers of health inequity [50].

This study has some limitations. The GBD study relies on modeling and estimation techniques, which may introduce some uncertainty into the reported findings. The GBD modeling techniques interpolate and extrapolate data where direct measurements are sparse or unavailable. The PAFs are calculated using the modeled exposure data and assumed effect sizes from the literature, which may not perfectly reflect the nuances of a particular local context, such as unique environmental factors, genetic predispositions, or social determinants of health. Therefore, the interpretation of the estimated attributable fractions should be approached cautiously, as they represent a probabilistic estimate rather than an exact causal relationship. This also implies that the GBD statistical estimates do not diminish the value of cancer registries, national health surveys, and well-designed research studies; rather, they can be used to supplement the evidence we can draw from such sources, especially when there is a lack of data. Some known and unknown modifiable risk factors, such as e-cigarette use, UV exposure, infections, and screening uptake, were not included in the GBD estimates, which may lead to an underreporting of the preventable burden of cancer in this study, particularly for some of the less prevalent/less studied cancers. Additionally, the results may not fully capture the complex interplay of multiple risk factors in cancer. Although the GBD modeling techniques address the interactive effects of risk factors (e.g., tobacco and alcohol, diet and obesity) using a hierarchical, counterfactual framework approach, the complexity of biological and social interactions means that this framework is a simplified representation of reality [19]. For example, some interactions may not be fully captured, or the data used to model the exposure-response relationships may have biases or be insufficient. Furthermore, there are no GBD subnational estimates for Australia, and hence, we were unable to further investigate disparities. Australia has a universal public health insurance scheme, Medicare, that funds public hospital care and provides subsidized access to private medical services. Although the system aims to provide all residents with access to high-quality cancer care, cancer outcomes vary across some population groups. Previous evidence shows significant disparities in cancer burden in Indigenous, remote, socioeconomically disadvantaged, and/or people from culturally and linguistically diverse (CALD) backgrounds [33,34,51,52]. Another limitation when analyzing risk-attributable cancer deaths is that advancements in cancer treatment may have reduced mortality. Notably, data from the Australian Institute of Health and Welfare [53] shows that between 2000 and 2024, the age-adjusted cancer mortality rate in Australia declined from 255 to 194 deaths per 100,000 people, while the age-adjusted cancer incidence rate rose from 582 to 624 cases per 100,000 people. Finally, while melanoma burden attributable to UV exposure was not included in this estimate, Australia has one of the highest rates of skin cancer in the world and UVR exposure is an established risk factor [54]. This is why public health campaigns in Australia, such as the “Slip, Slop, Slap, Seek, Slide” campaign, have been so focused on promoting sun protection [55]. Despite these limitations, this study provides valuable insights into the need for continued efforts to reduce the impact of modifiable risk factors on mortality and healthy life lost due to cancer. The results indicate that initiatives aimed at minimizing exposure to cancer risk factors at the population level would have a substantial impact on reducing the burden of cancer.

## 5. Conclusions

We provide a comprehensive examination of the contribution of modifiable risk factors to Australia’s cancer burden derived from GBD2021 data, which offers valuable insights for targeting risk reduction and cancer prevention strategies tailored to the Australian context. This study reinforces the need for continued efforts to address modifiable risk factors to reduce cancer burden and reiterates the importance of reliable, comprehensive cancer data in informing policy and practice.

## Figures and Tables

**Table 1:** List of GBD risks and cancer types included in the attributable cancer burden estimation of Australia in 2021.

Risk Factors	List of Cancers (ICD-10 Codes) ‡
0. All risks 1. Behavioral 1.1. Tobacco 1.1.1. Smoking 1.1.2. Second-hand smoke 1.1.3. Chewing tobacco 1.2. High alcohol use 1.3. Drug use 1.4. Dietary risks 1.4.1. Diet low in whole grains 1.4.2. Diet low in vegetables 1.4.3. Diet low in milk 1.4.4. Diet low in fruit 1.4.5. Diet low in fiber 1.4.6. Diet low in calcium 1.4.7. Diet high in sodium 1.4.8. Diet high in red meat 1.4.9. Diet high in processed meat 1.5. Low physical activity 1.6. Unsafe sex 2 Metabolic 2.1. High body mass index 2.2. High fasting plasma glucose 3. Environmental 3.1.1. Occupational carcinogens * 3.1.2. Particulate matter pollution 3.1.3. Residential radon	All cancers Tracheal, bronchus, and lung cancer (C33–C34) Lip and oral cavity cancer (C00–C06) Nasopharynx cancer (C11) Other pharynx cancer (C09–C10) Larynx cancer (C32) Thyroid cancer (C73) Esophageal cancer (C15) Stomach cancer (C16) Colon and rectum cancer (C18–C20) Liver cancer (C22) Gallbladder and biliary tract cancer (C23) Pancreatic cancer (C25) Breast cancer (C50) Cervical cancer (C53) Uterine cancer (C54) Ovarian cancer (C56) Prostate cancer (C61) Kidney cancer (C64) Bladder cancer (C67) Mesothelioma (C45) Non-Hodgkin lymphoma (C82–C86, C88) Multiple myeloma (C90) Leukemia (C91–C95)

Red highlighted box, list of risk factors and their associated levels; blue highlighted box, list of cancers and their ICD-10 codes. * Includes arsenic, asbestos, benzene, beryllium, cadmium, chromium, diesel engine exhaust, formaldehyde, nickel, polycyclic aromatic hydrocarbons, silica, sulfuric acid, and trichloroethylene. Each represents one GBD level 4 risk. ‡ Other cancer types such as malignant skin melanoma, non-melanoma skin cancer, testicular cancer, brain and central nervous system cancer, Hodgkin lymphoma, eye cancers, soft tissue and other extraosseous sarcomas, malignant neoplasm of bone and articular cartilage, and neuroblastoma and other peripheral nervous cell tumors were not included in the risk–cancer pairs and subsequent risk-attributable burden estimations either due to lack of sufficient data (data that is comparable and available across all countries in the GBD 2021 estimates) or absence of associated risk.

**Table 2:** Possible risk–cancer pairs estimated in the GBD 2021 for Australia.

Level-1 Risks	Level-2 Risks	Level-3 Risks	Cancer Type
**Behavioral risks**	Tobacco	Smoking	All cancers
			Tracheal, bronchus, and lung cancer
			Esophageal cancer
			Pancreatic cancer
			Colon and rectum cancer
			Liver cancer
			Leukemia
			Bladder cancer
			Larynx cancer
			Breast cancer
			Lip and oral cavity cancer
			Other pharynx cancer
			Cervical cancer
			Stomach cancer
			Kidney cancer
			Prostate cancer
			Nasopharynx cancer
		Second-hand smoke	Tracheal, bronchus, and lung cancer
			Breast cancer
		Chewing tobacco	Esophageal cancer
			Lip and oral cavity cancer
	Alcohol use	High alcohol use	All cancers
			Liver cancer
			Colon and rectum cancer
			Esophageal cancer
			Breast cancer
			Lip and oral cavity cancer
			Other pharynx cancer
			Nasopharynx cancer
			Larynx cancer
	Drug use		Liver cancer
	Dietary risks		All cancers
		Diet low in whole grains	Colon and rectum cancer
		Diet low in vegetables	Esophageal cancer
		Diet low in milk	Colon and rectum cancer
			Prostate cancer
		Diet low in fruit	Tracheal, bronchus, and lung cancer
		Diet low in fiber	Colon and rectum cancer
		Diet low in calcium	Colon and rectum cancer
			Prostate cancer
		Diet high in sodium	Stomach cancer
		Diet high in red meat	Colon and rectum cancer
			Breast cancer
		Diet high in processed meat	Colon and rectum cancer
	Low physical activity		Colon and rectum cancer
			Breast cancer
	Unsafe sex		Cervical cancer
**Metabolic risks**	High body mass index		All cancers
			Colon and rectum cancer
			Liver cancer
			Breast cancer
			Kidney cancer
			Leukemia
			Uterine cancer
			Ovarian cancer
			Non-Hodgkin lymphoma
			Pancreatic cancer
			Multiple myeloma
			Gallbladder and biliary tract cancer
			Thyroid cancer
	High fasting plasma glucose		All cancers
			Pancreatic cancer
			Colon and rectum cancer
			Tracheal, bronchus, and lung cancer
			Breast cancer
			Liver cancer
			Bladder cancer
**Environmental/** **Occupational** **risks**	Occupational risks	Occupational carcinogens	All cancers
			Tracheal, bronchus, and lung cancer
			Mesothelioma
			Ovarian cancer
			Larynx cancer
			Leukemia
	Air pollution	Particulate matter pollution	Tracheal, bronchus, and lung cancer
	Other environmental risks	Residential radon	Tracheal, bronchus, and lung cancer

Red highlighted box, Behavioral risk factors; blue highlighted box, metabolic risk factors; yellow highlighted box, environmental/occupational risks.

**Table 3:** Estimated cancer-related deaths and DALYs lost in Australia in 2021.

Cancer Type	Deaths			DALYs Lost		
	Both Sexes	Male	Female	Both Sexes	Male	Female
	No. (%)	No. (%)	No. (%)	No. (%)	No. (%)	No. (%)
**All cancers**	54,428 (31.1)	31,404 (33.8)	23,024 (27.9)	1,139,729 (17.0)	652,347 (19.2)	487,381 (14.8)
Bladder cancer	1399 (0.8)	991 (1.1)	408 (0.5)	22,833 (0.3)	16,534 (0.5)	6299 (0.2)
Brain and central nervous system cancer ‡	1657 (0.9)	1013 (1.1)	644 (0.8)	47,608 (0.7)	29,337 (0.9)	18,271 (0.6)
Breast cancer	3606 (2.1)	40 (<0.1)	3566 (4.3)	92,253 (1.4)	963 (<0.1)	91,289 (2.8)
Cervical cancer	-	-	406 (0.5)	-	-	11,420 (0.3)
Colon and rectum cancer	6674 (3.8)	3620 (3.9)	3054 (3.7)	134,818 (2)	76,352 (2.2)	58,465 (1.8)
Eye cancers ‡	48 (<0.1)	27 (<0.1)	21 (<0.1)	1187 (<0.1)	699 (<0.1)	488 (<0.1)
Gallbladder and biliary tract cancer	486 (0.3)	191 (0.2)	295 (0.4)	9114 (0.1)	3790 (0.1)	5323 (0.2)
Hodgkin lymphoma ‡	86 (<0.1)	53 (0.1)	33 (<0.1)	2668 (<0.1)	1695 (<0.1)	973 (<0.1)
Kidney cancer	1327 (0.8)	916 (1)	411 (0.5)	27,576 (0.4)	19,704 (0.6)	7872 (0.2)
Larynx cancer	234 (0.1)	203 (0.2)	31 (<0.1)	5114 (0.1)	4440 (0.1)	674 (<0.1)
Leukemia	2262 (1.3)	1392 (1.5)	870 (1.1)	46,552 (0.7)	28,853 (0.8)	17,699 (0.5)
Lip and oral cavity cancer	614 (0.4)	399 (0.4)	215 (0.3)	14,232 (0.2)	9777 (0.3)	4455 (0.1)
Liver cancer	2277 (1.3)	1552 (1.7)	725 (0.9)	51,025 (0.8)	35,835 (1.1)	15,190 (0.5)
Malignant neoplasm of bone and articular cartilage ‡	110 (0.1)	67 (0.1)	44 (0.1)	3766 (0.1)	2372 (0.1)	1394 (<0.1)
Malignant skin melanoma ‡	1715 (1)	1146 (1.2)	570 (0.7)	42,066 (0.6)	27,669 (0.8)	14,397 (0.4)
Mesothelioma	851 (0.5)	710 (0.8)	141 (0.2)	15,513 (0.2)	12,708 (0.4)	2806 (0.1)
Multiple myeloma	1378 (0.8)	793 (0.9)	585 (0.7)	26,582 (0.4)	15,677 (0.5)	10,905 (0.3)
Nasopharynx cancer	103 (0.1)	78 (0.1)	25 (<0.1)	2936 (<0.1)	2254 (0.1)	682 (<0.1)
Neuroblastoma and other peripheral nerve tumors ‡	26 (<0.1)	15 (<0.1)	11 (<0.1)	1194 (<0.1)	686 (<0.1)	508 (<0.1)
Non-Hodgkin lymphoma	2054 (1.2)	1185 (1.3)	868 (1.1)	42,770 (0.6)	25,692 (0.8)	17,078 (0.5)
Non-melanoma skin cancer ‡	800 (0.5)	550 (0.6)	250 (0.3)	14,081 (0.2)	10,364 (0.3)	3717 (0.1)
Esophageal cancer	1757 (1)	1276 (1.4)	481 (0.6)	35,247 (0.5)	27,180 (0.8)	8067 (0.2)
Other malignant neoplasms ‡	950 (0.5)	418 (0.5)	532 (0.6)	21,153 (0.3)	9757 (0.3)	11,396 (0.3)
Other neoplasms ‡	845 (0.5)	508 (0.5)	337 (0.4)	13,027 (0.2)	8124 (0.2)	4903 (0.1)
Other pharynx cancer	318 (0.2)	260 (0.3)	58 (0.1)	8317 (0.1)	6928 (0.2)	1389 (<0.1)
Ovarian cancer	-	-	1140 (1.4)	-	-	24,876 (0.8)
Pancreatic cancer	3700 (2.1)	1962 (2.1)	1738 (2.1)	72,186 (1.1)	39,927 (1.2)	32,259 (1)
Prostate cancer	-	4470 (4.8)	-	-	78,711 (2.3)	-
Soft tissue and other extraosseous sarcomas ‡	404 (0.2)	223 (0.2)	182 (0.2)	10,861 (0.2)	5995 (0.2)	4865 (0.1)
Stomach cancer	1796 (1)	1113 (1.2)	683 (0.8)	34,495 (0.5)	22,164 (0.7)	12,331 (0.4)
Testicular cancer ‡	-	35 (<0.1)	-	-	1996 (0.1)	-
Thyroid cancer	170 (0.1)	86 (0.1)	84 (0.1)	4318 (0.1)	2244 (0.1)	2074 (0.1)
Tracheal, bronchus, and lung cancer	10,246 (5.8)	6113 (6.6)	4133 (5)	208,320 (3.1)	123,919 (3.6)	84,401 (2.6)
Uterine cancer	-	-	485 (0.6)	-	-	10,916 (0.3)

‡ These cancers were not included in the subsequent risk-attributable burden estimations either due to a lack of sufficient data or the absence of associated risk. % represents the proportion of total deaths and DALYs in Australia in 2021 contributed by the specific cancer.

**Table 4:** Risk-attributable cancer deaths in Australia in 2021.

Risk	Cancer	Both Sexes			Males			Females		
		Number	% Attributable	ASR per Million	Number	% Attributable	ASR per Million	Number	% Attributable	ASR per Million
**All risk factors**	All cancers	20,409 (16,224, 24,146)	37.5 (30.6, 43.4)	438.9 (352.1, 516.4)	11,886 (9931, 13,594)	37.8 (32.3, 42.7)	548.7 (459, 626.1)	8523 (6289, 10,644)	37 (28, 44.9)	342.7 (256.9, 421.9)
	Bladder cancer	256 (154, 369)	18.3 (11.1, 25.5)	5.2 (3.2, 7.3)	189 (112, 268)	19 (11.5, 26.7)	8.4 (5.1, 12)	67 (38, 98)	16.5 (9.4, 23)	2.4 (1.4, 3.5)
	Breast cancer	1285 (540, 1928)	35.6 (14.6, 51.6)	28.8 (12.6, 42.6)	10 (4, 16)	24.1 (9.2, 38.1)	0.5 (0.2, 0.8)	1275 (532, 1914)	35.8 (14.6, 51.8)	53.9 (23.7, 79.5)
	Cervical cancer	-	-	-	-	-	-	406 (355, 449)	100	18.9 (16.7, 20.9)
	Colon and rectum cancer	4104 (2620, 5359)	61.5 (40.8, 76.9)	86.2 (55.4, 112.3)	2209 (1406, 2876)	61 (40.3, 76.4)	102.6 (65.4, 133.3)	1895 (1181, 2534)	62 (40, 77.5)	71.5 (45.6, 95.2)
	Gallbladder and biliary tract cancer	88 (58, 122)	18 (12.2, 24.4)	1.8 (1.2, 2.5)	33 (22, 47)	17.5 (11.7, 24.1)	1.5 (1, 2.1)	54 (36, 77)	18.4 (12.6, 24.8)	2 (1.4, 2.9)
	Kidney cancer	406 (204, 616)	30.6 (16.6, 45.1)	8.8 (4.5, 13.2)	282 (145, 424)	30.8 (16.9, 45.3)	13.3 (6.9, 19.9)	124 (59, 193)	30.2 (15.5, 44.3)	4.8 (2.3, 7.4)
	Larynx cancer	167 (140, 193)	71.3 (64.1, 77.6)	3.7 (3.1, 4.2)	148 (123, 172)	73 (65.5, 79.4)	6.9 (5.8, 8.1)	18 (14, 22)	59.9 (51.5, 68)	0.8 (0.6, 0.9)
	Leukemia	479 (328, 707)	21.2 (14.8, 30.4)	10 (7, 14.4)	313 (210, 468)	22.5 (15.3, 32.7)	14.2 (9.7, 21.1)	166 (112, 244)	19.1 (13.9, 26.4)	6.4 (4.4, 9.1)
	Lip and oral cavity cancer	287 (228, 346)	46.7 (39.2, 54.6)	6.5 (5.3, 7.8)	213 (170, 261)	53.3 (44.9, 61.3)	10.3 (8.2, 12.6)	74 (55, 93)	34.5 (27.2, 42.6)	3 (2.3, 3.7)
	Liver cancer	1621 (1246, 1946)	71.2 (55.5, 80.9)	36.6 (28.3, 43.6)	1202 (942, 1447)	77.4 (63.4, 85.8)	57.3 (45.3, 68.6)	419 (276, 551)	57.8 (38.5, 70.7)	17.8 (12, 23.2)
	Mesothelioma	840 (753, 907)	98.8 (98.5, 99.1)	17.2 (15.5, 18.5)	703 (639, 755)	99.1 (98.8, 99.4)	31.1 (28.4, 33.4)	137 (114, 155)	97.1 (96, 97.8)	5.4 (4.6, 6.1)
	Multiple myeloma	140 (−61, 351)	10.2 (−4.5, 25.4)	2.9 (−1.3, 7.3)	79 (−34, 199)	9.9 (−4.4, 25.1)	3.6 (−1.6, 9.1)	61 (−26, 157)	10.5 (−4.6, 26.2)	2.3 (−1, 6)
	Nasopharynx cancer	47 (34, 62)	45.9 (36.9, 54)	1.2 (0.9, 1.6)	39 (28, 52)	50.2 (40.7, 58.4)	2.1 (1.5, 2.7)	8 (6, 11)	32.8 (24.6, 40.5)	0.4 (0.3, 0.5)
	Non-Hodgkin lymphoma	145 (49, 257)	7 (2.3, 12.2)	3 (1, 5.4)	82 (28, 146)	6.9 (2.2, 12)	3.8 (1.3, 6.8)	63 (21, 110)	7.3 (2.4, 12.4)	2.4 (0.8, 4.2)
	Esophageal cancer	931 (633, 1171)	53 (36.7, 66)	20.1 (13.9, 25.2)	721 (514, 891)	56.5 (40.7, 69)	33.8 (24.4, 41.7)	210 (115, 292)	43.6 (24.4, 58.5)	7.8 (4.3, 10.7)
	Other pharynx cancer	173 (137, 209)	54.4 (45.7, 61.8)	4.1 (3.3, 5)	149 (119, 180)	57.4 (48.5, 65.2)	7.4 (5.9, 9)	24 (18, 29)	41.1 (32.9, 48.8)	1 (0.8, 1.3)
	Ovarian cancer	-	-	-	-	-	-	271 (160, 397)	23.8 (14, 33.9)	10.7 (6.1, 15.7)
	Pancreatic cancer	1223 (429, 1942)	33.1 (11.1, 53.1)	26 (9.5, 40.9)	677 (233, 1076)	34.5 (11.6, 55.4)	31.3 (11.2, 49.6)	546 (183, 890)	31.5 (10.7, 50.5)	21.3 (7.5, 34.4)
	Prostate cancer	-	-	-	49 (8, 108)	1.1 (0.2, 2.3)	2.2 (0.4, 4.8)	-	-	-
	Stomach cancer	201 (77, 701)	11.2 (4.4, 38.1)	4.4 (1.7, 14.9)	135 (53, 456)	12.1 (4.9, 40.1)	6.4 (2.5, 21.1)	66 (22, 240)	9.7 (3.4, 34.8)	2.6 (0.9, 9.3)
	Thyroid cancer	28 (20, 38)	16.4 (12.4, 20.2)	0.6 (0.4, 0.8)	14 (10, 19)	16.6 (12.5, 20.7)	0.7 (0.5, 0.9)	14 (9, 19)	16.3 (12.4, 20)	0.5 (0.4, 0.7)
	Tracheal, bronchus, and lung cancer	7060 (6090, 7962)	68.9 (63.3, 73.8)	150.9 (131.5, 169.4)	4640 (4050, 5240)	75.9 (69.2, 81.2)	211.2 (184.9, 238.3)	2420 (1993, 2809)	58.5 (52.1, 64.8)	98.6 (82.5, 113.3)
	Uterine cancer	-	-	-	-	-	-	203 (140, 270)	41.8 (30.4, 53.9)	8.1 (5.7, 10.8)
**Behavioral risks**	All cancers	13,600 (10,595, 16,214)	25 (19.7, 29.3)	298.4 (234.3, 353.3)	7818 (6331, 9196)	24.9 (20.6, 28.9)	366.2 (297.4, 430.2)	5782 (4182, 7147)	25.1 (18.5, 30.4)	237.2 (174.2, 289.2)
	Bladder cancer	177 (133, 235)	12.7 (9.7, 16.3)	3.6 (2.8, 4.8)	130 (98, 171)	13.1 (10, 17.2)	5.9 (4.5, 7.7)	47 (32, 66)	11.6 (8.4, 15.4)	1.7 (1.2, 2.3)
	Breast cancer	905 (372, 1443)	25.1 (10.2, 38.5)	20.9 (9, 32.5)	10 (4, 16)	24.1 (9.2, 38.1)	0.5 (0.2, 0.8)	896 (368, 1428)	25.1 (10.2, 38.5)	39.1 (17, 60.5)
	Cervical cancer	-	-	-	-	-	-	406 (355, 449)	100	18.9 (16.7, 20.9)
	Colon and rectum cancer	3444 (1740, 4799)	51.6 (27, 69.5)	72.4 (37.2, 100.5)	1840 (927, 2558)	50.8 (25.6, 68.7)	85.6 (43.1, 118.9)	1604 (804, 2280)	52.4 (28.1, 70.7)	60.6 (30.4, 85.7)
	Kidney cancer	86 (46, 145)	6.5 (3.4, 10.7)	1.9 (1, 3.1)	65 (34, 107)	7.1 (3.7, 11.5)	3.1 (1.6, 5)	22 (12, 37)	5.3 (2.8, 8.7)	0.8 (0.5, 1.4)
	Larynx cancer	149 (124, 175)	63.7 (55.1, 71.7)	3.3 (2.7, 3.9)	132 (108, 156)	64.9 (55.7, 72.9)	6.2 (5.1, 7.3)	17 (13, 21)	56.2 (47.4, 65)	0.7 (0.6, 0.9)
	Leukemia	227 (66, 457)	10 (2.8, 19.3)	4.5 (1.3, 9.1)	162 (44, 328)	11.6 (3.1, 22.4)	7.1 (2, 14.4)	66 (19, 133)	7.5 (2.3, 14.3)	2.4 (0.7, 4.8)
	Lip and oral cavity cancer	287 (228, 346)	46.7 (39.2, 54.6)	6.5 (5.3, 7.8)	213 (170, 261)	53.3 (44.9, 61.3)	10.3 (8.2, 12.6)	74 (55, 93)	34.5 (27.2, 42.6)	3 (2.3, 3.7)
	Liver cancer	1435 (1029, 1768)	63 (45.7, 73.6)	32.6 (23.5, 39.8)	1103 (839, 1366)	71.1 (54.8, 80.2)	52.6 (40.1, 64.5)	332 (192, 457)	45.8 (26.2, 59.1)	14.4 (8.4, 19.2)
	Nasopharynx cancer	47 (34, 62)	45.9 (36.8, 54)	1.2 (0.9, 1.6)	39 (28, 51)	50.1 (40.6, 58.3)	2.1 (1.5, 2.7)	8 (6, 11)	32.7 (24.4, 40.4)	0.4 (0.3, 0.5)
	Esophageal cancer	931 (633, 1171)	53 (36.7, 66)	20.1 (13.9, 25.2)	721 (514, 891)	56.5 (40.7, 69)	33.8 (24.4, 41.7)	210 (115, 292)	43.6 (24.4, 58.5)	7.8 (4.3, 10.7)
	Other pharynx cancer	173 (137, 209)	54.4 (45.7, 61.8)	4.1 (3.3, 5)	149 (119, 180)	57.4 (48.5, 65.2)	7.4 (5.9, 9)	24 (18, 29)	41.1 (32.9, 48.8)	1 (0.8, 1.3)
	Pancreatic cancer	306 (250, 374)	8.3 (6.9, 9.9)	6.9 (5.7, 8.4)	175 (143, 216)	8.9 (7.3, 10.7)	8.5 (7, 10.4)	131 (98, 166)	7.6 (6.1, 9.2)	5.5 (4.3, 6.9)
	Prostate cancer	-	-	-	49 (8, 108)	1.1 (0.2, 2.3)	2.2 (0.4, 4.8)	-	-	-
	Stomach cancer	201 (77, 701)	11.2 (4.4, 38.1)	4.4 (1.7, 14.9)	135 (53, 456)	12.1 (4.9, 40.1)	6.4 (2.5, 21.1)	66 (22, 240)	9.7 (3.4, 34.8)	2.6 (0.9, 9.3)
	Tracheal, bronchus, and lung cancer	4776 (4081, 5598)	46.6 (40.9, 52.5)	105 (89.9, 122.5)	2896 (2442, 3421)	47.4 (40.7, 53.2)	134.6 (113.9, 158.5)	1880 (1499, 2236)	45.5 (38.4, 52.1)	78.3 (63.5, 92.8)
**Tobacco**	All cancers	6801 (5415, 8300)	12.5 (10, 15.1)	150.4 (120.7, 182.9)	4263 (3356, 5260)	13.6 (10.7, 16.6)	199.5 (157.7, 244)	2539 (1958, 3165)	11 (8.6, 13.4)	106.4 (83.1, 131.5)
	Bladder cancer	177 (133, 235)	12.7 (9.7, 16.3)	3.6 (2.8, 4.8)	130 (98, 171)	13.1 (10, 17.2)	5.9 (4.5, 7.7)	47 (32, 66)	11.6 (8.4, 15.4)	1.7 (1.2, 2.3)
	Breast cancer	-	-	-	-	-	-	98 (62, 140)	2.8 (1.7, 3.8)	4.7 (3, 6.8)
	Cervical cancer	-	-	-	-	-	-	68 (39, 101)	16.8 (9.6, 25)	3.5 (2.1, 5.1)
	Colon and rectum cancer	176 (106, 266)	2.6 (1.6, 3.8)	4.1 (2.5, 6.1)	104 (62, 158)	2.9 (1.7, 4.2)	5.2 (3.1, 7.7)	72 (43, 109)	2.3 (1.4, 3.4)	3.1 (1.8, 4.7)
	Kidney cancer	86 (46, 145)	6.5 (3.4, 10.7)	1.9 (1, 3.1)	65 (34, 107)	7.1 (3.7, 11.5)	3.1 (1.6, 5)	22 (12, 37)	5.3 (2.8, 8.7)	0.8 (0.5, 1.4)
	Larynx cancer	126 (101, 153)	53.8 (45.1, 62.8)	2.8 (2.3, 3.4)	110 (88, 135)	54.3 (45.4, 63.4)	5.2 (4.2, 6.4)	16 (12, 20)	50.6 (41.9, 60.2)	0.6 (0.5, 0.8)
	Leukemia	227 (66, 457)	10 (2.8, 19.3)	4.5 (1.3, 9.1)	162 (44, 328)	11.6 (3.1, 22.4)	7.1 (2, 14.4)	66 (19, 133)	7.5 (2.3, 14.3)	2.4 (0.7, 4.8)
	Lip and oral cavity cancer	107 (68, 155)	17.4 (11.4, 24.8)	2.5 (1.6, 3.6)	74 (46, 108)	18.6 (12, 26.4)	3.7 (2.3, 5.3)	33 (19, 48)	15.2 (9.5, 22.1)	1.3 (0.8, 1.9)
	Liver cancer	164 (52, 295)	7.2 (2.3, 12.6)	3.9 (1.3, 7)	119 (37, 218)	7.7 (2.4, 13.4)	6 (1.9, 10.9)	45 (14, 82)	6.2 (2, 11)	2 (0.6, 3.6)
	Nasopharynx cancer	12 (7, 18)	11.5 (7.6, 17.2)	0.3 (0.2, 0.4)	10 (6, 15)	12.3 (8, 18.7)	0.5 (0.3, 0.8)	2 (1, 3)	8.9 (5.7, 13.2)	0.1 (0.1, 0.2)
	Esophageal cancer	395 (278, 531)	22.5 (16.1, 29.5)	8.5 (6, 11.3)	311 (219, 411)	24.4 (17.5, 31.7)	14.5 (10.3, 19.1)	84 (54, 119)	17.6 (12, 24.4)	3.1 (2.1, 4.4)
	Other pharynx cancer	83 (58, 110)	26 (18.9, 33.4)	2 (1.4, 2.6)	70 (49, 92)	26.9 (19.4, 34.8)	3.6 (2.5, 4.7)	13 (9, 17)	22.3 (16.1, 29.2)	0.6 (0.4, 0.8)
	Pancreatic cancer	306 (250, 374)	8.3 (6.9, 9.9)	6.9 (5.7, 8.4)	175 (143, 216)	8.9 (7.3, 10.7)	8.5 (7, 10.4)	131 (98, 166)	7.6 (6.1, 9.2)	5.5 (4.3, 6.9)
	Prostate cancer	-	-	-	61 (24, 116)	1.4 (0.6, 2.5)	2.7 (1.1, 5.2)	-	-	-
	Stomach cancer	98 (76, 127)	5.4 (4.3, 6.9)	2.2 (1.7, 2.8)	68 (51, 90)	6.1 (4.7, 7.7)	3.2 (2.4, 4.2)	30 (21, 40)	4.4 (3.3, 5.7)	1.2 (0.9, 1.6)
	Tracheal, bronchus, and lung cancer	4617 (3933, 5434)	45.1 (39.1, 51.1)	101.7 (86.9, 118.7)	2804 (2358, 3336)	45.9 (39.3, 52.1)	130.5 (109.8, 153.9)	1813 (1451, 2172)	43.9 (36.5, 50.7)	75.6 (61.4, 89.9)
*** Smoking***	All cancers	6663 (5363, 8105)	12.2 (9.9, 14.7)	147.3 (120.6, 178.4)	4176 (3334, 5088)	13.3 (10.6, 16.1)	195.5 (156.4, 238)	2487 (1934, 3094)	10.8 (8.6, 13.1)	104.1 (81.9, 127.9)
	Bladder cancer	177 (133, 235)	12.7 (9.7, 16.3)	3.6 (2.8, 4.8)	130 (98, 171)	13.1 (10, 17.2)	5.9 (4.5, 7.7)	47 (32, 66)	11.6 (8.4, 15.4)	1.7 (1.2, 2.3)
	Breast cancer	-	-	-	-	-	-	85 (61, 111)	2.4 (1.8, 3.1)	4.1 (3, 5.4)
	Cervical cancer	-	-	-	-	-	-	68 (39, 101)	16.8 (9.6, 25)	3.5 (2.1, 5.1)
	Colon and rectum cancer	176 (106, 266)	2.6 (1.6, 3.8)	4.1 (2.5, 6.1)	104 (62, 158)	2.9 (1.7, 4.2)	5.2 (3.1, 7.7)	72 (43, 109)	2.3 (1.4, 3.4)	3.1 (1.8, 4.7)
	Kidney cancer	86 (46, 145)	6.5 (3.4, 10.7)	1.9 (1, 3.1)	65 (34, 107)	7.1 (3.7, 11.5)	3.1 (1.6, 5)	22 (12, 37)	5.3 (2.8, 8.7)	0.8 (0.5, 1.4)
	Larynx cancer	126 (101, 153)	53.8 (45.1, 62.8)	2.8 (2.3, 3.4)	110 (88, 135)	54.3 (45.4, 63.4)	5.2 (4.2, 6.4)	16 (12, 20)	50.6 (41.9, 60.2)	0.6 (0.5, 0.8)
	Leukemia	227 (66, 457)	10 (2.8, 19.3)	4.5 (1.3, 9.1)	162 (44, 328)	11.6 (3.1, 22.4)	7.1 (2, 14.4)	66 (19, 133)	7.5 (2.3, 14.3)	2.4 (0.7, 4.8)
	Lip and oral cavity cancer	99 (60, 148)	16.2 (10.1, 23.7)	2.3 (1.4, 3.4)	70 (42, 104)	17.4 (10.8, 25.3)	3.4 (2.1, 5.1)	30 (17, 46)	13.8 (8.1, 21)	1.2 (0.7, 1.8)
	Liver cancer	164 (52, 295)	7.2 (2.3, 12.6)	3.9 (1.3, 7)	119 (37, 218)	7.7 (2.4, 13.4)	6 (1.9, 10.9)	45 (14, 82)	6.2 (2, 11)	2 (0.6, 3.6)
	Nasopharynx cancer	12 (7, 18)	11.5 (7.6, 17.2)	0.3 (0.2, 0.4)	10 (6, 15)	12.3 (8, 18.7)	0.5 (0.3, 0.8)	2 (1, 3)	8.9 (5.7, 13.2)	0.1 (0.1, 0.2)
	Esophageal cancer	383 (264, 518)	21.8 (15.5, 28.9)	8.3 (5.7, 11.1)	300 (207, 398)	23.5 (16.7, 31)	14 (9.8, 18.5)	83 (52, 117)	17.2 (11.6, 24.1)	3.1 (2, 4.3)
	Other pharynx cancer	83 (58, 110)	26 (18.9, 33.4)	2 (1.4, 2.6)	70 (49, 92)	26.9 (19.4, 34.8)	3.6 (2.5, 4.7)	13 (9, 17)	22.3 (16.1, 29.2)	0.6 (0.4, 0.8)
	Pancreatic cancer	306 (250, 374)	8.3 (6.9, 9.9)	6.9 (5.7, 8.4)	175 (143, 216)	8.9 (7.3, 10.7)	8.5 (7, 10.4)	131 (98, 166)	7.6 (6.1, 9.2)	5.5 (4.3, 6.9)
	Prostate cancer	-	-	-	61 (24, 116)	1.4 (0.6, 2.5)	2.7 (1.1, 5.2)	-	-	-
	Stomach cancer	98 (76, 127)	5.4 (4.3, 6.9)	2.2 (1.7, 2.8)	68 (51, 90)	6.1 (4.7, 7.7)	3.2 (2.4, 4.2)	30 (21, 40)	4.4 (3.3, 5.7)	1.2 (0.9, 1.6)
	Tracheal, bronchus, and lung cancer	4511 (3894, 5270)	44 (38.8, 49.2)	99.3 (86.5, 115.7)	2733 (2341, 3206)	44.7 (39, 50.2)	127.1 (109.1, 148.3)	1779 (1424, 2116)	43 (36.1, 49.7)	74.1 (60.3, 87.9)
*** Second-hand smoke***	Breast cancer	-	-	-	-	-	-	13 (−3, 30)	0.4 (−0.1, 0.8)	0.6 (−0.1, 1.5)
	Tracheal, bronchus, and lung cancer	205 (21, 453)	2 (0.2, 4.4)	4.7 (0.5, 10.3)	142 (15, 320)	2.3 (0.2, 5.1)	6.8 (0.7, 15.3)	63 (6, 142)	1.5 (0.1, 3.4)	2.8 (0.3, 6.3)
*** Chewing tobacco***	Lip and oral cavity cancer	9 (5, 14)	1.5 (0.9, 2.3)	0.2 (0.1, 0.3)	6 (2, 10)	1.4 (0.6, 2.5)	0.3 (0.1, 0.5)	3 (2, 7)	1.6 (0.8, 3)	0.1 (0.1, 0.2)
	Esophageal cancer	16 (9, 25)	0.9 (0.5, 1.4)	0.3 (0.2, 0.5)	14 (7, 24)	1.1 (0.6, 1.9)	0.6 (0.3, 1.1)	2 (1, 4)	0.4 (0.2, 0.7)	0.1 (0, 0.1)
**Alcohol use**	All cancers	2582 (2169, 2966)	4.7 (4.1, 5.4)	58.4 (50.1, 66.7)	1873 (1591, 2148)	6 (5.2, 6.8)	89.4 (76.8, 102.2)	709 (532, 872)	3.1 (2.5, 3.7)	30.5 (23.9, 36.8)
	Breast cancer	270 (183, 364)	7.5 (5.3, 10.1)	6.5 (4.6, 8.6)	5 (3, 7)	11.7 (7.6, 15.8)	0.2 (0.1, 0.3)	265 (180, 358)	7.4 (5.2, 10.1)	12.3 (8.6, 16.1)
	Colon and rectum cancer	572 (421, 739)	8.6 (6.5, 10.8)	12.5 (9.3, 16.1)	412 (298, 522)	11.4 (8.7, 14.3)	19.4 (14.2, 24.6)	160 (104, 227)	5.2 (3.7, 6.8)	6.5 (4.4, 9.2)
	Larynx cancer	52 (29, 75)	22.3 (11.9, 31.8)	1.2 (0.6, 1.7)	49 (27, 69)	23.9 (13, 33.8)	2.3 (1.3, 3.3)	4 (2, 6)	11.7 (5.3, 18)	0.2 (0.1, 0.2)
	Lip and oral cavity cancer	221 (164, 277)	36 (28.1, 43.7)	5 (3.8, 6.2)	172 (130, 218)	43.1 (34.1, 51.1)	8.3 (6.3, 10.5)	49 (33, 65)	23 (16.4, 30)	2 (1.4, 2.6)
	Liver cancer	890 (688, 1129)	39.1 (31.1, 47.3)	20.1 (15.6, 25)	744 (578, 940)	48 (38.6, 57.1)	35.4 (27.3, 44)	146 (100, 209)	20.1 (14.1, 27)	6.2 (4.3, 8.7)
	Nasopharynx cancer	40 (28, 55)	39 (29.3, 47.3)	1 (0.7, 1.4)	34 (23, 46)	43.1 (32.6, 51.7)	1.8 (1.3, 2.4)	7 (4, 9)	26.1 (18.3, 33.5)	0.3 (0.2, 0.5)
	Esophageal cancer	413 (296, 535)	23.5 (17, 30.3)	9.1 (6.6, 11.7)	348 (247, 445)	27.3 (19.7, 34.7)	16.5 (11.9, 21.1)	65 (42, 92)	13.5 (9, 18.6)	2.5 (1.7, 3.4)
	Other pharynx cancer	124 (90, 159)	39 (29.6, 47.2)	2.9 (2.1, 3.8)	110 (80, 139)	42.3 (32.2, 50.7)	5.5 (4, 6.9)	14 (10, 19)	24.6 (17.2, 32.1)	0.6 (0.4, 0.8)
**Drug use**	Liver cancer	485 (29, 767)	21.3 (1.3, 33.6)	11.1 (0.8, 17.1)	323 (24, 518)	20.8 (1.6, 32)	15.4 (1.4, 24.2)	162 (4, 275)	22.5 (0.6, 37.6)	7.2 (0.2, 11.9)
**Dietary risks**	All cancers	3884 (813, 6516)	7.1 (1.6, 12)	82.5 (17.3, 139.1)	1877 (409, 3072)	6 (1.3, 9.9)	87.2 (18.9, 142.7)	2007 (405, 3467)	8.7 (1.9, 15.1)	78.2 (15.6, 135.6)
	Breast cancer	493 (0, 1054)	13.7 (0, 29)	11.1 (0, 23.6)	6 (0, 12)	13.7 (0, 29)	0.3 (0, 0.6)	488 (0, 1042)	13.7 (0, 29)	20.6 (0, 43.7)
	Colon and rectum cancer	2638 (606, 4237)	39.5 (9.1, 62.1)	55.3 (12.6, 88.8)	1377 (274, 2251)	38 (7.6, 60.6)	63.9 (12.6, 104.4)	1261 (333, 2039)	41.2 (10.8, 63.5)	47.6 (12.3, 76.6)
	Esophageal cancer	369 (−83, 738)	21 (−4.8, 41.9)	7.8 (−1.8, 15.7)	268 (−58, 543)	21 (−4.7, 41.9)	12.5 (−2.7, 25.3)	101 (−25, 208)	20.9 (−4.8, 41.6)	3.7 (−0.9, 7.6)
	Prostate cancer	-	-	-	−13 (−31, 2)	−0.3 (−0.7, 0)	−0.6 (−1.3, 0.1)	-	-	-
	Stomach cancer	109 (0, 629)	6.1 (0, 34.8)	2.3 (0, 13.4)	71 (0, 406)	6.4 (0, 36)	3.4 (0, 18.9)	38 (0, 219)	5.5 (0, 31.9)	1.5 (0, 8.5)
	Tracheal, bronchus, and lung cancer	287 (146, 425)	2.8 (1.4, 4.1)	6.1 (3.1, 9.1)	168 (85, 255)	2.7 (1.4, 4)	7.7 (3.9, 11.7)	119 (58, 176)	2.9 (1.5, 4.2)	4.8 (2.4, 7)
*** Diet high in processed meat***	Colon and rectum cancer	611 (−143, 1282)	9.1 (−2.2, 18.6)	13 (−3.1, 27.2)	322 (−77, 671)	8.9 (−2.2, 18.2)	15.1 (−3.6, 31.5)	289 (−67, 618)	9.4 (−2.3, 19.2)	11.1 (−2.6, 23.7)
*** Diet high in red meat***	Breast cancer	493 (0, 1054)	13.7 (0, 29)	11.1 (0, 23.6)	6 (0, 12)	13.7 (0, 29)	0.3 (0, 0.6)	488 (0, 1042)	13.7 (0, 29)	20.6 (0, 43.7)
	Colon and rectum cancer	1135 (−1, 2271)	17 (0, 33.5)	23.9 (0, 47.6)	617 (−1, 1238)	17 (0, 33.5)	28.7 (0, 57.5)	518 (−1, 1047)	16.9 (0, 33.4)	19.6 (0, 39.5)
*** Diet high in sodium***	Stomach cancer	109 (0, 629)	6.1 (0, 34.8)	2.3 (0, 13.4)	71 (0, 406)	6.4 (0, 36)	3.4 (0, 18.9)	38 (0, 219)	5.5 (0, 31.9)	1.5 (0, 8.5)
*** Diet low in calcium***	Colon and rectum cancer	252 (165, 352)	3.8 (2.7, 5)	5.1 (3.4, 7.1)	52 (26, 88)	1.4 (0.7, 2.4)	2.4 (1.2, 4)	200 (129, 287)	6.5 (4.6, 8.7)	7.3 (4.7, 10.4)
	Prostate cancer	-	-	-	−13 (−31, 2)	−0.3 (−0.7, 0)	−0.6 (−1.3, 0.1)	-	-	-
*** Diet low in fiber***	Colon and rectum cancer	97 (43, 163)	1.5 (0.7, 2.3)	2 (0.9, 3.4)	46 (19, 80)	1.3 (0.5, 2.1)	2.1 (0.9, 3.6)	51 (21, 89)	1.7 (0.7, 2.7)	1.9 (0.8, 3.2)
*** Diet low in fruit***	Tracheal, bronchus, and lung cancer	287 (146, 425)	2.8 (1.4, 4.1)	6.1 (3.1, 9.1)	168 (85, 255)	2.7 (1.4, 4)	7.7 (3.9, 11.7)	119 (58, 176)	2.9 (1.5, 4.2)	4.8 (2.4, 7)
*** Diet low in milk***	Colon and rectum cancer	334 (79, 614)	5 (1.2, 8.9)	6.8 (1.6, 12.4)	48 (10, 110)	1.3 (0.3, 3)	2.2 (0.5, 5.1)	286 (70, 513)	9.3 (2.3, 16.6)	10.6 (2.6, 18.9)
*** Diet low in vegetables***	Esophageal cancer	369 (−83, 738)	21 (−4.8, 41.9)	7.8 (−1.8, 15.7)	268 (−58, 543)	21 (−4.7, 41.9)	12.5 (−2.7, 25.3)	101 (−25, 208)	20.9 (−4.8, 41.6)	3.7 (−0.9, 7.6)
*** Diet low in whole grains***	Colon and rectum cancer	1189 (483, 1810)	17.8 (7.4, 26.6)	24.9 (10.3, 37.9)	649 (271, 984)	17.9 (7.5, 26.7)	30.1 (12.6, 45.6)	540 (214, 846)	17.7 (7.3, 26.4)	20.4 (8.1, 31.7)
**Low physical activity**	Breast cancer	-	-	-	-	-	-	130 (25, 238)	3.6 (0.7, 6.5)	5.3 (1, 9.6)
	Colon and rectum cancer	668 (396, 964)	10 (6.1, 14.4)	13.4 (8.1, 19.2)	283 (148, 443)	7.8 (4.2, 11.9)	12.8 (6.7, 19.9)	385 (228, 581)	12.6 (7.8, 18.1)	13.9 (8.5, 20.9)
**Unsafe sex**	Cervical cancer	-	-	-	-	-	-	406 (355, 449)	100	18.9 (16.7, 20.9)
**Metabolic risks**	All cancers	5102 (1483, 8768)	9.4 (2.7, 15.8)	108.2 (31.5, 185.4)	2479 (829, 4165)	7.9 (2.6, 13.2)	114.9 (38.8, 192.4)	2623 (664, 4517)	11.4 (2.9, 19.6)	102.2 (25.9, 175.3)
	Bladder cancer	90 (−12, 196)	6.4 (−0.9, 13.9)	1.7 (−0.2, 3.8)	67 (−9, 147)	6.8 (−1, 14.8)	3 (−0.4, 6.5)	23 (−3, 51)	5.6 (−0.8, 11.7)	0.8 (−0.1, 1.7)
	Breast cancer	-	-	-	-	-	-	509 (−66, 1054)	14.3 (−1.8, 28.3)	20 (−2.7, 41)
	Colon and rectum cancer	1378 (655, 2075)	20.7 (9.8, 30.8)	28.9 (13.8, 43.5)	758 (366, 1155)	21 (10, 31.2)	35.1 (16.9, 53.5)	620 (287, 956)	20.3 (9.6, 30.2)	23.4 (10.8, 36)
	Gallbladder and biliary tract cancer	88 (58, 122)	18 (12.2, 24.4)	1.8 (1.2, 2.5)	33 (22, 47)	17.5 (11.7, 24.1)	1.5 (1, 2.1)	54 (36, 77)	18.4 (12.6, 24.8)	2 (1.4, 2.9)
	Kidney cancer	342 (141, 559)	25.8 (10.6, 41.1)	7.4 (3, 12)	234 (97, 379)	25.5 (10.5, 40.3)	11 (4.5, 17.8)	108 (42, 178)	26.3 (10.8, 41.5)	4.2 (1.6, 6.8)
	Leukemia	273 (197, 360)	12.1 (9, 15.3)	5.8 (4.2, 7.6)	168 (123, 221)	12 (8.9, 15.4)	7.8 (5.7, 10.2)	106 (72, 143)	12.2 (9.1, 15.3)	4.1 (2.9, 5.5)
	Liver cancer	487 (187, 828)	21.4 (8.4, 35.9)	11 (4.2, 18.7)	324 (121, 552)	20.9 (8.2, 35.2)	15.6 (5.9, 26.6)	163 (62, 273)	22.5 (8.6, 37.3)	6.8 (2.6, 11.2)
	Multiple myeloma	140 (−61, 351)	10.2 (−4.5, 25.4)	2.9 (−1.3, 7.3)	79 (−34, 199)	9.9 (−4.4, 25.1)	3.6 (−1.6, 9.1)	61 (−26, 157)	10.5 (−4.6, 26.2)	2.3 (−1, 6)
	Non-Hodgkin lymphoma	145 (49, 257)	7 (2.3, 12.2)	3 (1, 5.4)	82 (28, 146)	6.9 (2.2, 12)	3.8 (1.3, 6.8)	63 (21, 110)	7.3 (2.4, 12.4)	2.4 (0.8, 4.2)
	Ovarian cancer	-	-	-	-	-	-	151 (39, 274)	13.2 (3.5, 23.2)	6.3 (1.7, 11.3)
	Pancreatic cancer	999 (125, 1781)	27 (3.3, 48.4)	20.9 (2.6, 37.2)	550 (68, 993)	28.1 (3.4, 50.7)	25.1 (3.1, 45.2)	449 (57, 828)	25.9 (3.2, 46.4)	17.2 (2.2, 31.5)
	Thyroid cancer	28 (20, 38)	16.4 (12.4, 20.2)	0.6 (0.4, 0.8)	14 (10, 19)	16.6 (12.5, 20.7)	0.7 (0.5, 0.9)	14 (9, 19)	16.3 (12.4, 20)	0.5 (0.4, 0.7)
	Tracheal, bronchus, and lung cancer	270 (−54, 585)	2.6 (−0.6, 5.7)	5.7 (−1.1, 12.3)	170 (−33, 370)	2.8 (−0.6, 6)	7.7 (−1.5, 16.7)	100 (−21, 218)	2.4 (−0.5, 5.2)	4 (−0.8, 8.6)
	Uterine cancer	-	-	-	-	-	-	203 (140, 270)	41.8 (30.4, 53.9)	8.1 (5.7, 10.8)
**High body mass index**	All cancers	3203 (1235, 5346)	5.9 (2.3, 9.7)	68.7 (26.6, 114.3)	1454 (610, 2394)	4.6 (1.9, 7.5)	68.4 (28.8, 112.1)	1749 (598, 2948)	7.6 (2.6, 12.6)	68.7 (23.7, 115.5)
	Breast cancer	-	-	-	-	-	-	360 (−11, 723)	10.1 (−0.3, 19.6)	13.9 (−0.4, 27.6)
	Colon and rectum cancer	916 (395, 1454)	13.7 (5.9, 21.7)	19.4 (8.4, 30.9)	492 (214, 775)	13.6 (5.7, 21.5)	23 (10, 36.3)	424 (181, 682)	13.9 (6.1, 21.7)	16.3 (7, 26.2)
	Gallbladder and biliary tract cancer	88 (58, 122)	18 (12.2, 24.4)	1.8 (1.2, 2.5)	33 (22, 47)	17.5 (11.7, 24.1)	1.5 (1, 2.1)	54 (36, 77)	18.4 (12.6, 24.8)	2 (1.4, 2.9)
	Kidney cancer	342 (141, 559)	25.8 (10.6, 41.1)	7.4 (3, 12)	234 (97, 379)	25.5 (10.5, 40.3)	11 (4.5, 17.8)	108 (42, 178)	26.3 (10.8, 41.5)	4.2 (1.6, 6.8)
	Leukemia	273 (197, 360)	12.1 (9, 15.3)	5.8 (4.2, 7.6)	168 (123, 221)	12 (8.9, 15.4)	7.8 (5.7, 10.2)	106 (72, 143)	12.2 (9.1, 15.3)	4.1 (2.9, 5.5)
	Liver cancer	419 (174, 716)	18.4 (7.8, 30.6)	9.5 (4, 16.2)	286 (115, 489)	18.4 (7.6, 31)	13.8 (5.6, 23.6)	133 (57, 222)	18.4 (7.8, 29.8)	5.6 (2.4, 9.3)
	Multiple myeloma	140 (−61, 351)	10.2 (−4.5, 25.4)	2.9 (−1.3, 7.3)	79 (−34, 199)	9.9 (−4.4, 25.1)	3.6 (−1.6, 9.1)	61 (−26, 157)	10.5 (−4.6, 26.2)	2.3 (−1, 6)
	Non-Hodgkin lymphoma	145 (49, 257)	7 (2.3, 12.2)	3 (1, 5.4)	82 (28, 146)	6.9 (2.2, 12)	3.8 (1.3, 6.8)	63 (21, 110)	7.3 (2.4, 12.4)	2.4 (0.8, 4.2)
	Ovarian cancer	-	-	-	-	-	-	151 (39, 274)	13.2 (3.5, 23.2)	6.3 (1.7, 11.3)
	Pancreatic cancer	139 (−3, 333)	3.8 (−0.1, 9.3)	3 (−0.1, 7.2)	67 (−4, 168)	3.4 (−0.2, 8.8)	3.2 (−0.2, 7.8)	71 (0, 166)	4.1 (0, 9.7)	2.9 (0, 6.5)
	Thyroid cancer	28 (20, 38)	16.4 (12.4, 20.2)	0.6 (0.4, 0.8)	14 (10, 19)	16.6 (12.5, 20.7)	0.7 (0.5, 0.9)	14 (9, 19)	16.3 (12.4, 20)	0.5 (0.4, 0.7)
	Uterine cancer	-	-	-	-	-	-	203 (140, 270)	41.8 (30.4, 53.9)	8.1 (5.7, 10.8)
**High fasting plasma glucose**	All cancers	2048 (266, 3639)	3.8 (0.5, 6.8)	42.7 (5.4, 75.6)	1093 (182, 1950)	3.5 (0.6, 6.2)	49.6 (8.3, 88.9)	955 (84, 1743)	4.1 (0.4, 7.6)	36.6 (2.9, 67.4)
	Bladder cancer	90 (−12, 196)	6.4 (−0.9, 13.9)	1.7 (−0.2, 3.8)	67 (−9, 147)	6.8 (−1, 14.8)	3 (−0.4, 6.5)	23 (−3, 51)	5.6 (−0.8, 11.7)	0.8 (−0.1, 1.7)
	Breast cancer	-	-	-	-	-	-	173 (−53, 391)	4.9 (−1.4, 11.1)	7 (−2.2, 15.8)
	Colon and rectum cancer	541 (285, 798)	8.1 (4.3, 11.9)	11.1 (5.8, 16.4)	311 (160, 467)	8.6 (4.4, 12.9)	14.2 (7.3, 21.3)	229 (119, 351)	7.5 (3.8, 11)	8.5 (4.4, 12.8)
	Liver cancer	73 (9, 138)	3.2 (0.4, 6)	1.6 (0.2, 3)	41 (4, 80)	2.6 (0.3, 5)	1.9 (0.2, 3.7)	32 (4, 66)	4.4 (0.6, 8.6)	1.3 (0.2, 2.5)
	Pancreatic cancer	902 (114, 1581)	24.4 (3.1, 43.5)	18.8 (2.4, 32.9)	504 (64, 909)	25.7 (3.2, 45.9)	22.9 (2.9, 41.2)	397 (50, 725)	22.9 (3, 40.7)	15.1 (1.9, 27.7)
	Tracheal, bronchus, and lung cancer	270 (−54, 585)	2.6 (−0.6, 5.7)	5.7 (−1.1, 12.3)	170 (−33, 370)	2.8 (−0.6, 6)	7.7 (−1.5, 16.7)	100 (−21, 218)	2.4 (−0.5, 5.2)	4 (−0.8, 8.6)
**Environmental/** **occupational risks**	All cancers	5040 (4113, 5877)	9.3 (7.8, 10.5)	103.1 (84.1, 120.5)	3872 (3140, 4561)	12.3 (10, 14.3)	171.1 (138.5, 201.7)	1168 (839, 1478)	5.1 (4.1, 6.1)	44.8 (32.9, 56.4)
	Larynx cancer	46 (28, 66)	19.7 (12, 26.9)	0.9 (0.6, 1.3)	44 (26, 63)	21.4 (12.7, 29.9)	1.9 (1.1, 2.8)	2 (1, 4)	7.9 (4.2, 12)	0.1 (0, 0.1)
	Leukemia	8 (2, 14)	0.4 (0.1, 0.6)	0.2 (0.1, 0.4)	4 (1, 8)	0.3 (0.1, 0.5)	0.2 (0.1, 0.4)	4 (1, 7)	0.4 (0.1, 0.7)	0.2 (0.1, 0.4)
	Mesothelioma	840 (753, 907)	98.8 (98.5, 99.1)	17.2 (15.5, 18.5)	703 (639, 755)	99.1 (98.8, 99.4)	31.1 (28.4, 33.4)	137 (114, 155)	97.1 (96, 97.8)	5.4 (4.6, 6.1)
	Ovarian cancer	-	-	-	-	-	-	138 (66, 220)	12.1 (6.1, 19)	4.9 (2.4, 7.8)
	Tracheal, bronchus, and lung cancer	4007 (3116, 4817)	39.1 (31.4, 45.4)	82.1 (63.5, 98.5)	3120 (2402, 3819)	51 (39.7, 60.2)	137.9 (105.9, 168.9)	887 (599, 1158)	21.4 (15.9, 27)	34.1 (23.9, 44.4)
*** Occupational carcinogens***	All cancers	4594 (3654, 5406)	8.4 (6.9, 9.7)	93.3 (74.8, 109.5)	3658 (2894, 4354)	11.6 (9.2, 13.7)	161 (127.2, 191.5)	936 (656, 1214)	4.1 (3.1, 5.1)	35.3 (25.1, 45.5)
	Larynx cancer	46 (28, 66)	19.7 (12, 26.9)	0.9 (0.6, 1.3)	44 (26, 63)	21.4 (12.7, 29.9)	1.9 (1.1, 2.8)	2 (1, 4)	7.9 (4.2, 12)	0.1 (0, 0.1)
	Leukemia	8 (2, 14)	0.4 (0.1, 0.6)	0.2 (0.1, 0.4)	4 (1, 8)	0.3 (0.1, 0.5)	0.2 (0.1, 0.4)	4 (1, 7)	0.4 (0.1, 0.7)	0.2 (0.1, 0.4)
	Mesothelioma	840 (753, 907)	98.8 (98.5, 99.1)	17.2 (15.5, 18.5)	703 (639, 755)	99.1 (98.8, 99.4)	31.1 (28.4, 33.4)	137 (114, 155)	97.1 (96, 97.8)	5.4 (4.6, 6.1)
	Ovarian cancer	-	-	-	-	-	-	138 (66, 220)	12.1 (6.1, 19)	4.9 (2.4, 7.8)
	Tracheal, bronchus, and lung cancer	3562 (2695, 4293)	34.7 (27.2, 41.2)	72.3 (54.3, 87)	2907 (2154, 3604)	47.5 (36.1, 57.3)	127.7 (94, 158.3)	655 (437, 902)	15.8 (11, 21.1)	24.7 (16.5, 33.7)
*** Particulate matter pollution***	Tracheal, bronchus, and lung cancer	571 (280, 914)	5.6 (2.9, 8.8)	12.2 (6, 19.6)	341 (167, 547)	5.6 (2.9, 8.8)	15.6 (7.7, 25)	230 (113, 364)	5.6 (2.9, 8.8)	9.3 (4.6, 14.6)
*** Residential radon***	Tracheal, bronchus, and lung cancer	118 (−51, 423)	1.2 (−0.5, 4.2)	2.5 (−1.1, 9.1)	71 (−30, 256)	1.2 (−0.5, 4.2)	3.2 (−1.4, 11.7)	48 (−20, 173)	1.2 (−0.5, 4.2)	1.9 (−0.8, 7)

**Risk column**: Bold = GBD level-1 risks, italics = GBD level-3 risks, other GBD level-2 risks. The “All cancers” category excludes non-melanoma skin cancer. ASR, age-standardized death rate. Numbers in brackets represent 95% uncertainty intervals (UI). % denotes proportion of deaths from the specific cancer attributable to the risk factor. Malignant skin melanoma, non-melanoma skin cancer, testicular cancer, brain and central nervous system cancer, Hodgkin lymphoma, hepatoblastoma, eye cancers, soft tissue and other extraosseous sarcomas, malignant neoplasm of bone and articular cartilage, and neuroblastoma and other peripheral nervous cell tumors were not estimated either due to lack of data or absence of associated risk.

**Table 5:** Risk-attributable cancer DALYs lost in Australia in 2021.

Risk	Cancer	Both Sexes			Males			Females		
		Number	% Attributable	ASR per Million	Number	% Attributable	ASR per Million	Number	% Attributable	ASR per Million
**All risk factors**	All cancers	431,575 (351,199, 503,322)	37.9 (31, 43.7)	10,175.6 (8269.9, 11,799.9)	250,154 (210,905, 282,954)	38.3 (32.7, 43.1)	12,235.9 (10,302.1, 13,829.9)	181,420 (138,884, 220,846)	37.2 (28.1, 44.8)	8312.5 (6386.5, 10,029.8)
	Bladder cancer	4707 (3100, 6450)	20.6 (13.7, 27.7)	102.8 (68.9, 138.5)	3555 (2306, 4856)	21.5 (14.4, 29)	166.2 (110, 223)	1153 (702, 1625)	18.3 (11.6, 24.5)	46.7 (29.5, 64.3)
	Breast cancer	32,797 (15,088, 48,267)	35.6 (16.1, 51)	825.8 (394.4, 1204.3)	244 (96, 391)	25.2 (10.6, 38.9)	12.5 (5, 20)	32,553 (14,918, 47,916)	35.7 (16.2, 51.1)	1579.4 (755.9, 2301.2)
	Cervical cancer	-	-	-	-	-	-	11,419 (10,264, 12,624)	100	628.4 (567.7, 695.2)
	Colon and rectum cancer	83,568 (53,641, 107,793)	62 (41.3, 77.3)	1957.1 (1255.5, 2522.4)	47,118 (30,545, 60,407)	61.7 (41.1, 77)	2338.4 (1517.7, 2994.3)	36,450 (23,307, 47,967)	62.3 (40.5, 77.7)	1606.4 (1019.5, 2116.9)
	Gallbladder and biliary tract cancer	1704 (1149, 2359)	18.7 (12.8, 25.2)	39.1 (26.3, 53.9)	692 (464, 946)	18.3 (12.2, 25)	34.1 (22.9, 46.7)	1012 (679, 1396)	19 (13, 25.4)	43.4 (29.3, 59.5)
	Kidney cancer	8713 (4440, 13,011)	31.6 (17.1, 46.3)	208.2 (106.8, 310.3)	6292 (3289, 9327)	31.9 (17.7, 46.5)	318.1 (166.1, 471.2)	2421 (1146, 3714)	30.7 (15.9, 44.9)	106.7 (50.6, 161.8)
	Larynx cancer	3763 (3157, 4324)	73.6 (66.7, 79.1)	89.4 (74.8, 102.9)	3340 (2807, 3879)	75.2 (68.1, 80.8)	165.6 (138.9, 191.7)	422 (341, 509)	62.7 (54.8, 70.3)	19.2 (15.5, 23.2)
	Leukemia	9207 (6684, 12,952)	19.8 (14.3, 27)	213.6 (158.5, 292.3)	6064 (4269, 8800)	21 (14.9, 29.6)	295.2 (212.3, 418.7)	3143 (2240, 4410)	17.7 (13.5, 23.4)	141 (104.4, 188.1)
	Lip and oral cavity cancer	7231 (5917, 8649)	50.8 (43.3, 58.5)	180.2 (148.5, 214.7)	5529 (4480, 6761)	56.5 (49, 64.5)	287.5 (234.7, 349.4)	1702 (1317, 2090)	38.2 (30.6, 45.9)	80.1 (61.9, 98.2)
	Liver cancer	37,330 (29,062, 43,994)	73.2 (57.8, 81.9)	913.2 (716.3, 1072)	27,954 (22,383, 33,106)	78 (64.7, 85.7)	1417.2 (1141.8, 1671.5)	9376 (6342, 12,010)	61.8 (41.3, 73.5)	444.2 (302.5, 561.7)
	Mesothelioma	15,110 (13,797, 16,250)	97.4 (96.8, 98)	328.3 (300.3, 352.6)	12,463 (11,430, 13,375)	98.1 (97.4, 98.6)	569.8 (523.4, 610.4)	2647 (2266, 2956)	94.3 (92.5, 95.7)	114.7 (99.1, 127.8)
	Multiple myeloma	2809 (−1289, 7084)	10.6 (−4.8, 26.3)	63.4 (−29.3, 159.4)	1631 (−752, 4085)	10.4 (−4.7, 26.2)	78.3 (−36.2, 196.2)	1178 (−533, 2961)	10.8 (−4.9, 26.8)	50 (−22.9, 125.7)
	Nasopharynx cancer	1415 (1024, 1879)	48.2 (38.8, 56.5)	39.7 (28.7, 53.1)	1172 (854, 1545)	52 (42.7, 60.2)	67.4 (49, 89)	243 (169, 337)	35.6 (26.7, 44.1)	13.6 (9.3, 19)
	Non-Hodgkin lymphoma	3095 (1043, 5557)	7.2 (2.3, 12.5)	73.1 (24.4, 130.9)	1823 (623, 3272)	7.1 (2.3, 12.4)	91.8 (31.2, 164.7)	1272 (421, 2213)	7.4 (2.4, 12.6)	56 (18.2, 96.9)
	Esophageal cancer	19,286 (13,556, 24,124)	54.7 (38.7, 66.7)	454.3 (323.3, 564)	15,672 (11,464, 19,220)	57.7 (42.5, 69.3)	780.9 (575.1, 953.6)	3614 (2088, 4931)	44.8 (26.2, 59.2)	151.3 (90, 204.9)
	Other pharynx cancer	4813 (3869, 5819)	57.9 (49.6, 65.3)	123.3 (99.5, 148.1)	4187 (3370, 5051)	60.4 (52.2, 67.9)	221.8 (179.2, 267.5)	626 (481, 763)	45.1 (36.3, 52.8)	30.6 (24.1, 37.1)
	Ovarian cancer	-	-	-	-	-	-	5300 (2867, 7967)	21.3 (11.6, 31.3)	233.7 (119.8, 358.4)
	Pancreatic cancer	24,414 (9440, 37,874)	33.8 (12.7, 53)	562.5 (228.8, 862.1)	14,069 (5489, 22,017)	35.3 (13.5, 55.4)	689 (283.1, 1063.3)	10,344 (3844, 16,455)	32.1 (11.7, 50.5)	445.2 (174, 697.3)
	Prostate cancer	-	-	-	1157 (326, 2266)	1.5 (0.4, 2.7)	54.4 (16, 104.8)	-	-	-
	Stomach cancer	4151 (1704, 13,762)	12 (5.1, 39.3)	100.9 (42.1, 327.6)	2856 (1173, 9200)	12.9 (5.5, 41.1)	145.5 (60, 460)	1295 (486, 4539)	10.5 (4.1, 36.3)	60.1 (23.4, 208.9)
	Thyroid cancer	722 (503, 953)	16.7 (12.7, 20.5)	18 (12.6, 23.8)	379 (272, 500)	16.9 (12.8, 20.9)	20.1 (14.4, 26.5)	343 (233, 468)	16.5 (12.7, 20.4)	16.1 (11, 22)
	Tracheal, bronchus, and lung cancer	144,177 (127,821, 160,906)	69.2 (64.1, 73.9)	3301.6 (2936.7, 3668.3)	93,958 (82,945, 105,036)	75.8 (69.9, 80.8)	4482.3 (3968.1, 4994.9)	50,219 (42,351, 57,280)	59.5 (53.3, 65.4)	2235.2 (1912.7, 2544.6)
	Uterine cancer	-	-	-	-	-	-	4688 (3310, 6207)	43 (31.3, 54.9)	210.8 (149.7, 278.7)
**Behavioral risks**	All cancers	302,471 (238,505, 356,803)	26.5 (21.2, 30.8)	7289.8 (5777.1, 8563.5)	174,434 (142,659, 202,439)	26.7 (22.3, 30.8)	8680.4 (7119.3, 10,035.2)	128,036 (95,543, 155,068)	26.3 (19.6, 31.6)	6012 (4529.8, 7274.9)
	Bladder cancer	3468 (2750, 4446)	15.2 (12.2, 18.9)	77.5 (62.2, 97.6)	2617 (2059, 3338)	15.8 (12.6, 19.9)	124.1 (98.5, 155.7)	851 (622, 1123)	13.5 (10.4, 17)	35.4 (26.6, 46.3)
	Breast cancer	24,395 (10,939, 37,193)	26.4 (11.9, 39.8)	637.6 (298.6, 965.2)	244 (96, 391)	25.2 (10.6, 38.9)	12.5 (5, 20)	24,152 (10,856, 36,858)	26.5 (11.9, 39.8)	1220.6 (574.2, 1847.1)
	Cervical cancer	-	-	-	-	-	-	11,419 (10,264, 12,624)	100	628.4 (567.7, 695.2)
	Colon and rectum cancer	70,218 (36,275, 97,190)	52 (27, 69.8)	1648.4 (844, 2263.2)	39,346 (19,991, 53,806)	51.5 (26.3, 69.2)	1957.6 (992.1, 2676.4)	30,872 (15,929, 43,196)	52.7 (27.5, 71)	1363.5 (698, 1900)
	Kidney cancer	1905 (1057, 3039)	6.9 (3.8, 10.8)	45.3 (25.6, 71.3)	1482 (821, 2364)	7.5 (4.1, 11.7)	74.4 (41.7, 117)	423 (242, 691)	5.4 (3, 8.5)	18.4 (10.6, 29.3)
	Larynx cancer	3470 (2888, 4024)	67.8 (59.9, 74.5)	83.3 (69.3, 96.6)	3067 (2525, 3588)	69.1 (60.9, 75.8)	153.4 (126.3, 179.8)	403 (324, 487)	59.8 (51.5, 67.4)	18.4 (14.9, 22.2)
	Leukemia	3993 (1211, 7804)	8.6 (2.5, 16)	85.7 (26.7, 164.7)	2905 (863, 5778)	10.1 (2.9, 19.1)	133.1 (40.3, 263.1)	1088 (344, 2174)	6.1 (1.9, 11.3)	43.6 (14.2, 85.3)
	Lip and oral cavity cancer	7231 (5917, 8649)	50.8 (43.3, 58.5)	180.2 (148.5, 214.7)	5529 (4480, 6761)	56.5 (49, 64.5)	287.5 (234.7, 349.4)	1702 (1317, 2090)	38.2 (30.6, 45.9)	80.1 (61.9, 98.2)
	Liver cancer	33,434 (24,419, 40,383)	65.5 (48.7, 74.8)	819.4 (592.9, 984.1)	25,716 (19,814, 31,401)	71.8 (57, 79.9)	1302.1 (1010.2, 1587.6)	7718 (4526, 10,151)	50.9 (29.8, 63.6)	370.5 (215.7, 481.1)
	Nasopharynx cancer	1412 (1022, 1875)	48.1 (38.7, 56.4)	39.7 (28.6, 53)	1170 (852, 1542)	51.9 (42.5, 60.1)	67.2 (48.9, 88.8)	242 (168, 336)	35.4 (26.6, 44)	13.5 (9.2, 18.9)
	Esophageal cancer	19,286 (13,556, 24,124)	54.7 (38.7, 66.7)	454.3 (323.3, 564)	15,672 (11,464, 19,220)	57.7 (42.5, 69.3)	780.9 (575.1, 953.6)	3614 (2088, 4931)	44.8 (26.2, 59.2)	151.3 (90, 204.9)
	Other pharynx cancer	4813 (3869, 5819)	57.9 (49.6, 65.3)	123.3 (99.5, 148.1)	4187 (3370, 5051)	60.4 (52.2, 67.9)	221.8 (179.2, 267.5)	626 (481, 763)	45.1 (36.3, 52.8)	30.6 (24.1, 37.1)
	Pancreatic cancer	7091 (5993, 8357)	9.8 (8.5, 11.4)	175.2 (148.8, 206.8)	4222 (3541, 5082)	10.6 (9, 12.2)	219.7 (185.5, 262.6)	2869 (2241, 3533)	8.9 (7.3, 10.7)	133.1 (106.1, 163.3)
	Prostate cancer	-	-	-	1157 (326, 2266)	1.5 (0.4, 2.7)	54.4 (16, 104.8)	-	-	-
	Stomach cancer	4151 (1704, 13,762)	12 (5.1, 39.3)	100.9 (42.1, 327.6)	2856 (1173, 9200)	12.9 (5.5, 41.1)	145.5 (60, 460)	1295 (486, 4539)	10.5 (4.1, 36.3)	60.1 (23.4, 208.9)
	Tracheal, bronchus, and lung cancer	105,027 (90,578, 121,265)	50.4 (44.7, 56.3)	2470 (2130.6, 2848.7)	64,266 (55,174, 74,644)	51.9 (45.3, 57.6)	3146.1 (2724.5, 3638.5)	40,761 (33,751, 47,972)	48.3 (41.8, 54.8)	1844.5 (1549.3, 2163.6)
Tobacco	All cancers	152,765 (123,710, 185,128)	13.4 (10.8, 16.1)	3649.8 (2962.6, 4413.9)	96,267 (77,163, 117,369)	14.8 (11.9, 17.7)	4767.8 (3842, 5775.3)	56,497 (44,733, 69,777)	11.6 (9.3, 14)	2617.2 (2098.7, 3218.4)
	Bladder cancer	3468 (2750, 4446)	15.2 (12.2, 18.9)	77.5 (62.2, 97.6)	2617 (2059, 3338)	15.8 (12.6, 19.9)	124.1 (98.5, 155.7)	851 (622, 1123)	13.5 (10.4, 17)	35.4 (26.6, 46.3)
	Breast cancer	3125 (1951, 4493)	3.4 (2.1, 4.8)	86.8 (53.2, 124.8)	6 (−1, 15)	0.6 (−0.1, 1.4)	0.3 (−0.1, 0.8)	3118 (1951, 4482)	3.4 (2.2, 4.8)	168.5 (103.3, 242.1)
	Cervical cancer	-	-	-	-	-	-	2249 (1337, 3291)	19.7 (11.7, 28.7)	129.7 (77.3, 189.4)
	Colon and rectum cancer	4513 (2775, 6702)	3.3 (2, 4.7)	116 (71.3, 170.9)	2750 (1659, 4038)	3.6 (2.2, 5.1)	146.6 (88.3, 214.7)	1763 (1054, 2657)	3 (1.8, 4.3)	87.2 (52.6, 130.6)
	Kidney cancer	1905 (1057, 3039)	6.9 (3.8, 10.8)	45.3 (25.6, 71.3)	1482 (821, 2364)	7.5 (4.1, 11.7)	74.4 (41.7, 117)	423 (242, 691)	5.4 (3, 8.5)	18.4 (10.6, 29.3)
	Larynx cancer	2984 (2427, 3546)	58.4 (50.2, 66.2)	72 (58.6, 85.3)	2620 (2121, 3139)	59 (50.6, 67)	131.6 (106.3, 157.3)	364 (289, 450)	54.1 (45.7, 62.7)	16.6 (13.2, 20.4)
	Leukemia	3993 (1211, 7804)	8.6 (2.5, 16)	85.7 (26.7, 164.7)	2905 (863, 5778)	10.1 (2.9, 19.1)	133.1 (40.3, 263.1)	1088 (344, 2174)	6.1 (1.9, 11.3)	43.6 (14.2, 85.3)
	Lip and oral cavity cancer	2820 (1853, 4082)	19.8 (13.1, 27.5)	70.4 (46.4, 101.3)	2060 (1299, 2998)	21.1 (14, 29.2)	107.7 (68.5, 155.6)	760 (487, 1101)	17 (11, 23.9)	35.1 (23, 49.4)
	Liver cancer	4171 (1374, 7342)	8.2 (2.7, 14.2)	107.5 (35.7, 188.3)	3101 (1015, 5500)	8.6 (2.8, 15.1)	165.5 (55.1, 292.3)	1070 (340, 1967)	7 (2.3, 12.4)	52.7 (16.7, 96.7)
	Nasopharynx cancer	332 (208, 513)	11.3 (7.9, 16.2)	8.9 (5.7, 13.5)	273 (171, 420)	12.1 (8.3, 17.4)	15.1 (9.5, 22.7)	59 (37, 89)	8.7 (5.8, 12.3)	3.1 (1.9, 4.6)
	Esophageal cancer	8207 (5932, 10,692)	23.3 (17.1, 29.9)	191.9 (141.2, 248.3)	6751 (4837, 8707)	24.8 (18.2, 31.9)	333.3 (241.4, 430.8)	1456 (989, 2017)	18.1 (13, 24.4)	60.6 (42.9, 82.3)
	Other pharynx cancer	2415 (1717, 3148)	29 (21.8, 36.5)	63 (45, 81.6)	2067 (1466, 2702)	29.8 (22.2, 37.8)	111.4 (79.3, 144.6)	348 (248, 455)	25.1 (18.4, 31.9)	17.1 (12.3, 22.4)
	Pancreatic cancer	7091 (5993, 8357)	9.8 (8.5, 11.4)	175.2 (148.8, 206.8)	4222 (3541, 5082)	10.6 (9, 12.2)	219.7 (185.5, 262.6)	2869 (2241, 3533)	8.9 (7.3, 10.7)	133.1 (106.1, 163.3)
	Prostate cancer	-	-	-	1359 (572, 2395)	1.7 (0.8, 2.9)	63.3 (26.7, 110.8)	-	-	-
	Stomach cancer	2091 (1663, 2620)	6.1 (4.9, 7.4)	51.2 (40.9, 62.7)	1477 (1140, 1885)	6.7 (5.4, 8.2)	75.5 (58.4, 95.7)	613 (473, 777)	5 (4, 6.2)	28.9 (22.8, 36)
	Tracheal, bronchus, and lung cancer	102,042 (87,632, 118,895)	49 (43.2, 55.1)	2402.4 (2076.6, 2781.7)	62,578 (53,431, 73,113)	50.5 (44, 56.4)	3066.1 (2636.3, 3555.9)	39,463 (32,264, 46,697)	46.8 (39.9, 53.3)	1787.3 (1484.1, 2103.8)
*** Smoking***	All cancers	149,615 (122,749, 180,367)	13.1 (10.8, 15.6)	3572 (2937.8, 4290)	94,389 (76,500, 114,240)	14.5 (11.8, 17.2)	4673.8 (3812.2, 5637.7)	55,226 (44,456, 67,632)	11.3 (9.2, 13.6)	2553.8 (2075, 3114.9)
	Bladder cancer	3468 (2750, 4446)	15.2 (12.2, 18.9)	77.5 (62.2, 97.6)	2617 (2059, 3338)	15.8 (12.6, 19.9)	124.1 (98.5, 155.7)	851 (622, 1123)	13.5 (10.4, 17)	35.4 (26.6, 46.3)
	Breast cancer	-	-	-	-	-	-	2707 (1970, 3521)	3 (2.2, 3.8)	146.1 (106, 191.5)
	Cervical cancer	-	-	-	-	-	-	2249 (1337, 3291)	19.7 (11.7, 28.7)	129.7 (77.3, 189.4)
	Colon and rectum cancer	4513 (2775, 6702)	3.3 (2, 4.7)	116 (71.3, 170.9)	2750 (1659, 4038)	3.6 (2.2, 5.1)	146.6 (88.3, 214.7)	1763 (1054, 2657)	3 (1.8, 4.3)	87.2 (52.6, 130.6)
	Kidney cancer	1905 (1057, 3039)	6.9 (3.8, 10.8)	45.3 (25.6, 71.3)	1482 (821, 2364)	7.5 (4.1, 11.7)	74.4 (41.7, 117)	423 (242, 691)	5.4 (3, 8.5)	18.4 (10.6, 29.3)
	Larynx cancer	2984 (2427, 3546)	58.4 (50.2, 66.2)	72 (58.6, 85.3)	2620 (2121, 3139)	59 (50.6, 67)	131.6 (106.3, 157.3)	364 (289, 450)	54.1 (45.7, 62.7)	16.6 (13.2, 20.4)
	Leukemia	3993 (1211, 7804)	8.6 (2.5, 16)	85.7 (26.7, 164.7)	2905 (863, 5778)	10.1 (2.9, 19.1)	133.1 (40.3, 263.1)	1088 (344, 2174)	6.1 (1.9, 11.3)	43.6 (14.2, 85.3)
	Lip and oral cavity cancer	2657 (1649, 3915)	18.7 (11.9, 26.3)	66.6 (41.9, 97.4)	1957 (1209, 2891)	20 (12.8, 28.3)	102.6 (63.7, 150.3)	700 (430, 1039)	15.7 (9.8, 23.1)	32.5 (20.4, 47.1)
	Liver cancer	4171 (1374, 7342)	8.2 (2.7, 14.2)	107.5 (35.7, 188.3)	3101 (1015, 5500)	8.6 (2.8, 15.1)	165.5 (55.1, 292.3)	1070 (340, 1967)	7 (2.3, 12.4)	52.7 (16.7, 96.7)
	Nasopharynx cancer	332 (208, 513)	11.3 (7.9, 16.2)	8.9 (5.7, 13.5)	273 (171, 420)	12.1 (8.3, 17.4)	15.1 (9.5, 22.7)	59 (37, 89)	8.7 (5.8, 12.3)	3.1 (1.9, 4.6)
	Esophageal cancer	7968 (5635, 10,483)	22.6 (16.5, 29.3)	186.3 (134.5, 243.2)	6536 (4638, 8497)	24.1 (17.5, 31.2)	322.7 (231.8, 419.8)	1432 (962, 1999)	17.8 (12.6, 24)	59.6 (41.9, 81.6)
	Other pharynx cancer	2415 (1717, 3148)	29 (21.8, 36.5)	63 (45, 81.6)	2067 (1466, 2702)	29.8 (22.2, 37.8)	111.4 (79.3, 144.6)	348 (248, 455)	25.1 (18.4, 31.9)	17.1 (12.3, 22.4)
	Pancreatic cancer	7091 (5993, 8357)	9.8 (8.5, 11.4)	175.2 (148.8, 206.8)	4222 (3541, 5082)	10.6 (9, 12.2)	219.7 (185.5, 262.6)	2869 (2241, 3533)	8.9 (7.3, 10.7)	133.1 (106.1, 163.3)
	Prostate cancer	-	-	-	1359 (572, 2395)	1.7 (0.8, 2.9)	63.3 (26.7, 110.8)	-	-	-
	Stomach cancer	2091 (1663, 2620)	6.1 (4.9, 7.4)	51.2 (40.9, 62.7)	1477 (1140, 1885)	6.7 (5.4, 8.2)	75.5 (58.4, 95.7)	613 (473, 777)	5 (4, 6.2)	28.9 (22.8, 36)
	Tracheal, bronchus, and lung cancer	99,711 (87,133, 115,111)	47.9 (42.9, 53.2)	2345.6 (2058.3, 2699.9)	61,025 (52,985, 70,395)	49.3 (43.7, 54.3)	2988.1 (2620.9, 3423.7)	38,686 (31,992, 45,530)	45.8 (39.7, 52.2)	1749.9 (1468.6, 2038.2)
* Second-hand smoke*	Breast cancer	433 (−97, 977)	0.5 (−0.1, 1)	12.2 (−2.8, 27.3)	6 (−1, 15)	0.6 (−0.1, 1.4)	0.3 (−0.1, 0.8)	427 (−96, 965)	0.5 (−0.1, 1)	23.4 (−5.3, 52.5)
	Tracheal, bronchus, and lung cancer	4870 (491, 10,619)	2.3 (0.2, 5)	120.6 (12.2, 258.9)	3368 (340, 7434)	2.7 (0.3, 5.9)	172.1 (17.4, 376.8)	1502 (140, 3418)	1.8 (0.2, 4)	72.9 (6.9, 165.1)
* Chewing tobacco*	Lip and oral cavity cancer	199 (121, 310)	0 (0, 0.1)	12 (7.8, 17.2)	129 (55, 233)	1.3 (0.6, 2.4)	6.5 (2.8, 11.7)	70 (36, 119)	1.6 (0.8, 2.7)	3.1 (1.6, 5.3)
	Esophageal cancer	312 (177, 502)	1.4 (0.8, 2.2)	4.7 (2.9, 7.4)	283 (157, 474)	1 (0.6, 1.7)	13.9 (7.7, 22.9)	29 (14, 54)	0.4 (0.2, 0.6)	1.2 (0.6, 2.2)
Alcohol use	All cancers	61,981 (53,555, 70,886)	5.4 (4.7, 6.2)	1542.7 (1342.4, 1768.5)	44,294 (38,709, 50,418)	6.8 (5.9, 7.7)	2259.4 (1991.5, 2565.8)	17,686 (13,942, 21,390)	3.6 (2.9, 4.3)	879.9 (702.9, 1057.6)
	Breast cancer	8053 (5729, 10,491)	8.7 (6.4, 11.4)	219.6 (159, 283.2)	123 (80, 173)	12.8 (8.7, 17)	6.4 (4.2, 9)	7929 (5622, 10,323)	8.7 (6.3, 11.3)	420.3 (304.6, 542.4)
	Colon and rectum cancer	12,910 (9806, 16,460)	9.6 (7.5, 11.9)	315.1 (242.8, 400)	9397 (7087, 11,739)	12.3 (9.7, 15.1)	477.3 (363.8, 597.2)	3513 (2420, 4909)	6 (4.3, 7.9)	166.7 (115.8, 233.1)
	Larynx cancer	1205 (655, 1690)	23.6 (13.4, 33)	29 (15.9, 40.5)	1119 (614, 1571)	25.2 (14.3, 35.1)	56.1 (30.9, 78.3)	86 (38, 133)	12.8 (5.9, 19.3)	4 (1.8, 6.2)
	Lip and oral cavity cancer	5565 (4319, 6912)	39.1 (31.1, 46.8)	139.3 (107.9, 172.5)	4422 (3406, 5576)	45.2 (36.8, 53.2)	230.1 (178.3, 288.6)	1143 (813, 1448)	25.7 (18.9, 32.4)	54.7 (39, 68.8)
	Liver cancer	20,565 (15,936, 25,557)	40.3 (31.9, 48.7)	499.9 (389.1, 621.9)	17,334 (13,608, 21,667)	48.4 (38.7, 57.5)	873.8 (687.3, 1094.6)	3232 (2235, 4452)	21.3 (15.4, 28.2)	151.5 (106, 206.4)
	Nasopharynx cancer	1220 (879, 1688)	41.6 (31.8, 50)	34.6 (24.6, 47.8)	1021 (736, 1397)	45.3 (35.3, 54)	59.1 (42.5, 81)	200 (128, 292)	29.3 (20.6, 37.4)	11.4 (7.2, 16.7)
	Esophageal cancer	9037 (6678, 11,503)	25.6 (19.3, 32.6)	217.8 (162.3, 276.4)	7829 (5704, 9897)	28.8 (21.6, 36.3)	395.5 (292.6, 497.9)	1208 (829, 1647)	15 (10.4, 20.3)	52.8 (36.9, 71.4)
	Other pharynx cancer	3425 (2532, 4347)	41.2 (31.6, 49.6)	87.5 (65.6, 110.5)	3050 (2247, 3859)	44 (34.2, 52.6)	161 (120, 203.5)	375 (264, 496)	27.1 (18.6, 35)	18.5 (13.1, 24.3)
Drug use	Liver cancer	11,403 (965, 17,241)	22.4 (1.9, 33.3)	281.7 (29.8, 419)	7439 (785, 11,512)	20.7 (2.3, 30.7)	377 (48.8, 571.2)	3963 (152, 6324)	26.1 (1, 41.8)	194 (10.5, 299.6)
Dietary risks	All cancers	81,307 (16,452, 138,154)	7.1 (1.5, 12.2)	1930.8 (384.3, 3310.8)	39,769 (8369, 65,474)	6.1 (1.3, 10.2)	1972.6 (411.1, 3254.1)	41,537 (7977, 72,722)	8.5 (1.7, 15)	1893.8 (351, 3363)
	Breast cancer	12,608 (−7, 26,829)	13.7 (0, 28.9)	321.7 (−0.2, 686.6)	133 (0, 295)	13.7 (0, 29)	6.7 (0, 14.9)	12,476 (−7, 26,547)	13.7 (0, 28.9)	613.8 (−0.4, 1309.5)
	Colon and rectum cancer	53,560 (11,898, 85,652)	39.7 (8.7, 62.5)	1254.8 (275.6, 2006.2)	29,313 (5708, 47,205)	38.3 (7.4, 61.1)	1455.5 (280.5, 2342.7)	24,247 (6190, 38,891)	41.4 (10.4, 63.8)	1069.7 (269.8, 1702.5)
	Esophageal cancer	7337 (−1635, 14,786)	20.8 (−4.7, 41.5)	170.6 (−38.2, 345.4)	5663 (−1235, 11,416)	20.8 (−4.7, 41.7)	280.5 (−61.6, 565.2)	1674 (−406, 3426)	20.7 (−4.7, 41.2)	68.9 (−16.5, 140.2)
	Prostate cancer	-	-	-	−205 (−494, 32)	−0.3 (−0.6, 0)	−9.1 (−21.8, 1.4)	-	-	-
	Stomach cancer	2198 (0, 12,383)	6.4 (0, 35.7)	53.2 (0, 297.1)	1479 (0, 8216)	6.7 (0, 36.6)	75.2 (0, 411.5)	719 (0, 4173)	5.8 (0, 33.1)	33.1 (0, 191.3)
	Tracheal, bronchus, and lung cancer	5809 (3019, 8613)	2.8 (1.4, 4.1)	134.5 (69.8, 199)	3387 (1697, 5096)	2.7 (1.4, 4)	163.7 (82.5, 248.3)	2422 (1196, 3530)	2.9 (1.5, 4.2)	108.4 (54.1, 159.2)
* Diet high in processed meat*	Colon and rectum cancer	12,901 (−3072, 27,041)	9.5 (−2.4, 19.4)	306.8 (−73.6, 643.2)	7113 (−1714, 14,840)	9.3 (−2.3, 19)	356.8 (−86.3, 742.4)	5789 (−1373, 12,175)	9.9 (−2.4, 20)	260.5 (−62.4, 545.9)
* Diet high in red meat*	Breast cancer	12,608 (−7, 26,829)	13.7 (0, 28.9)	321.7 (−0.2, 686.6)	133 (0, 295)	13.7 (0, 29)	6.7 (0, 14.9)	12,476 (−7, 26,547)	13.7 (0, 28.9)	613.8 (−0.4, 1309.5)
	Colon and rectum cancer	23,235 (−23, 46,324)	17.2 (0, 33.9)	545.9 (−0.5, 1087.2)	13,177 (−11, 26,220)	17.2 (0, 33.8)	655.1 (−0.5, 1301.8)	10,057 (−9, 20,155)	17.2 (0, 33.8)	445.9 (−0.4, 890.8)
* Diet high in sodium*	Stomach cancer	2198 (0, 12,383)	6.4 (0, 35.7)	53.2 (0, 297.1)	1479 (0, 8216)	6.7 (0, 36.6)	75.2 (0, 411.5)	719 (0, 4173)	5.8 (0, 33.1)	33.1 (0, 191.3)
* Diet low in calcium*	Colon and rectum cancer	4595 (3025, 6498)	3.4 (2.4, 4.6)	104.3 (69.3, 146.9)	1011 (499, 1696)	1.3 (0.7, 2.2)	49.1 (24.2, 82.6)	3584 (2323, 5102)	6.1 (4.3, 8.2)	153 (97.3, 216.4)
	Prostate cancer	-	-	-	−205 (−494, 32)	−0.3 (−0.6, 0)	−9.1 (−21.8, 1.4)	-	-	-
* Diet low in fiber*	Colon and rectum cancer	2027 (926, 3352)	1.5 (0.7, 2.4)	47.6 (21.3, 77.5)	1058 (448, 1789)	1.4 (0.6, 2.2)	52.9 (21.8, 89.4)	969 (419, 1642)	1.7 (0.7, 2.7)	42.2 (18.7, 69.8)
* Diet low in fruit*	Tracheal, bronchus, and lung cancer	5809 (3019, 8613)	2.8 (1.4, 4.1)	134.5 (69.8, 199)	3387 (1697, 5096)	2.7 (1.4, 4)	163.7 (82.5, 248.3)	2422 (1196, 3530)	2.9 (1.5, 4.2)	108.4 (54.1, 159.2)
* Diet low in milk*	Colon and rectum cancer	6302 (1539, 11,382)	4.7 (1.2, 8.3)	146.5 (35.9, 262.1)	939 (202, 2101)	1.2 (0.3, 2.7)	47.6 (10.4, 102)	5364 (1299, 9476)	9.2 (2.3, 16.1)	235.1 (56.4, 411)
* Diet low in vegetables*	Esophageal cancer	7337 (−1635, 14,786)	20.8 (−4.7, 41.5)	170.6 (−38.2, 345.4)	5663 (−1235, 11,416)	20.8 (−4.7, 41.7)	280.5 (−61.6, 565.2)	1674 (−406, 3426)	20.7 (−4.7, 41.2)	68.9 (−16.5, 140.2)
* Diet low in whole grains*	Colon and rectum cancer	24,068 (10,149, 36,689)	17.8 (7.4, 26.7)	562.6 (240.5, 854.1)	13,709 (5798, 20,593)	17.9 (7.5, 26.8)	678.9 (287.2, 1019.3)	10,359 (4248, 16,090)	17.7 (7.3, 26.4)	456.3 (191.1, 704.8)
Low physical activity	Breast cancer	-	-	-	-	-	-	3129 (636, 5667)	3.4 (0.7, 6.1)	149.3 (31.2, 267)
	Colon and rectum cancer	12,065 (7517, 17,145)	8.9 (5.5, 12.5)	267.7 (164.5, 384.7)	5308 (2878, 8080)	7 (3.9, 10.4)	251.5 (138.2, 383.3)	6757 (4101, 9930)	11.6 (7.3, 16)	282.1 (172.3, 404.7)
Unsafe sex	Cervical cancer	-	-	-	-	-	-	11,419 (10,264, 12,624)	100	628.4 (567.7, 695.2)
**Metabolic risks**	All cancers	105,916 (30,902, 180,597)	9.3 (2.7, 15.6)	2459.1 (729.4, 4161.7)	52,513 (17,824, 87,860)	8.1 (2.7, 13.4)	2587.2 (896.2, 4307.6)	53,403 (13,465, 91,587)	11 (2.8, 18.7)	2340 (600.5, 4002.1)
	Bladder cancer	1460 (−200, 3122)	6.4 (−0.9, 13.6)	30.2 (−4.2, 64.6)	1112 (−151, 2372)	6.7 (−0.9, 14.5)	50.1 (−6.9, 107.2)	348 (−49, 746)	5.5 (−0.8, 11.5)	13.2 (−1.9, 28.3)
	Breast cancer	-	-	-	-	-	-	11,375 (−1585, 23,489)	12.5 (−1.7, 24.7)	488.2 (−72.5, 1002.9)
	Colon and rectum cancer	28,162 (13,270, 42,460)	20.9 (9.8, 31.1)	654.1 (307.4, 990.5)	16,200 (7839, 24,811)	21.2 (10, 31.6)	797.3 (385, 1215.4)	11,962 (5565, 18,305)	20.5 (9.7, 30.7)	522.5 (240.7, 799.1)
	Gallbladder and biliary tract cancer	1704 (1149, 2359)	18.7 (12.8, 25.2)	39.1 (26.3, 53.9)	692 (464, 946)	18.3 (12.2, 25)	34.1 (22.9, 46.7)	1012 (679, 1396)	19 (13, 25.4)	43.4 (29.3, 59.5)
	Kidney cancer	7323 (3009, 11,814)	26.5 (11, 41.9)	175.2 (71.6, 280.6)	5210 (2127, 8397)	26.4 (11, 41.7)	264 (107.4, 422.7)	2114 (843, 3400)	26.8 (11.1, 42.1)	93.4 (37.7, 149.6)
	Leukemia	5478 (4080, 7048)	11.8 (8.8, 14.9)	131.2 (99.2, 167.9)	3403 (2525, 4423)	11.8 (8.7, 15)	171.2 (127.9, 220.9)	2075 (1491, 2721)	11.7 (8.8, 14.7)	95.3 (69.4, 124.2)
	Liver cancer	11,208 (4275, 18,971)	22 (8.7, 36.4)	274.9 (105.2, 464.4)	7731 (2950, 13,233)	21.5 (8.6, 36.1)	396 (152.2, 675.1)	3476 (1333, 5734)	22.9 (8.8, 37.1)	161.4 (61.7, 265.9)
	Multiple myeloma	2809 (−1289, 7084)	10.6 (−4.8, 26.3)	63.4 (−29.3, 159.4)	1631 (−752, 4085)	10.4 (−4.7, 26.2)	78.3 (−36.2, 196.2)	1178 (−533, 2961)	10.8 (−4.9, 26.8)	50 (−22.9, 125.7)
	Non-Hodgkin lymphoma	3095 (1043, 5557)	7.2 (2.3, 12.5)	73.1 (24.4, 130.9)	1823 (623, 3272)	7.1 (2.3, 12.4)	91.8 (31.2, 164.7)	1272 (421, 2213)	7.4 (2.4, 12.6)	56 (18.2, 96.9)
	Ovarian cancer	-	-	-	-	-	-	3397 (908, 6081)	13.7 (3.6, 23.7)	160.8 (42.8, 287)
	Pancreatic cancer	19,159 (2378, 34,120)	26.6 (3.2, 47.5)	431.5 (53.1, 769.1)	10,972 (1337, 19,842)	27.5 (3.3, 49.8)	526.2 (63.7, 954.7)	8188 (1038, 14,932)	25.4 (3.1, 45.4)	344.1 (43.1, 626.2)
	Thyroid cancer	722 (503, 953)	16.7 (12.7, 20.5)	18 (12.6, 23.8)	379 (272, 500)	16.9 (12.8, 20.9)	20.1 (14.4, 26.5)	343 (233, 468)	16.5 (12.7, 20.4)	16.1 (11, 22)
	Tracheal, bronchus, and lung cancer	5336 (−1080, 11,682)	2.6 (−0.5, 5.6)	119.7 (−24.2, 260.1)	3361 (−649, 7282)	2.7 (−0.5, 5.8)	158.2 (−30.5, 342.6)	1975 (−411, 4309)	2.3 (−0.5, 5.1)	84.9 (−17.5, 185.7)
	Uterine cancer	-	-	-	-	-	-	4688 (3310, 6207)	43 (31.3, 54.9)	210.8 (149.7, 278.7)
High body mass index	All cancers	69,055 (26,844, 114,737)	6.1 (2.3, 9.9)	1629.3 (644.9, 2689)	32,413 (13,688, 53,245)	5 (2.1, 8.2)	1633.7 (696.8, 2664.9)	36,643 (12,683, 61,684)	7.5 (2.6, 12.3)	1621 (572.1, 2694.9)
	Breast cancer	-	-	-	-	-	-	7807 (−260, 15,384)	8.5 (−0.3, 16.5)	320.8 (−12.2, 628.3)
	Colon and rectum cancer	19,250 (8365, 30,518)	14.3 (6.2, 22.5)	454.6 (198.2, 718.5)	10,830 (4719, 17,156)	14.2 (6, 22.5)	540.9 (236.4, 855.7)	8420 (3657, 13,524)	14.4 (6.3, 22.3)	374.8 (162.6, 598.5)
	Gallbladder and biliary tract cancer	1704 (1149, 2359)	18.7 (12.8, 25.2)	39.1 (26.3, 53.9)	692 (464, 946)	18.3 (12.2, 25)	34.1 (22.9, 46.7)	1012 (679, 1396)	19 (13, 25.4)	43.4 (29.3, 59.5)
	Kidney cancer	7323 (3009, 11,814)	26.5 (11, 41.9)	175.2 (71.6, 280.6)	5210 (2127, 8397)	26.4 (11, 41.7)	264 (107.4, 422.7)	2114 (843, 3400)	26.8 (11.1, 42.1)	93.4 (37.7, 149.6)
	Leukemia	5478 (4080, 7048)	11.8 (8.8, 14.9)	131.2 (99.2, 167.9)	3403 (2525, 4423)	11.8 (8.7, 15)	171.2 (127.9, 220.9)	2075 (1491, 2721)	11.7 (8.8, 14.7)	95.3 (69.4, 124.2)
	Liver cancer	9835 (4101, 16,643)	19.3 (8.2, 31.8)	243.4 (101.2, 407.9)	6926 (2820, 11,803)	19.3 (8.2, 32.3)	357.1 (146.3, 609.5)	2909 (1213, 4803)	19.2 (8, 30.8)	136.8 (56.7, 224.1)
	Multiple myeloma	2809 (−1289, 7084)	10.6 (−4.8, 26.3)	63.4 (−29.3, 159.4)	1631 (−752, 4085)	10.4 (−4.7, 26.2)	78.3 (−36.2, 196.2)	1178 (−533, 2961)	10.8 (−4.9, 26.8)	50 (−22.9, 125.7)
	Non-Hodgkin lymphoma	3095 (1043, 5557)	7.2 (2.3, 12.5)	73.1 (24.4, 130.9)	1823 (623, 3272)	7.1 (2.3, 12.4)	91.8 (31.2, 164.7)	1272 (421, 2213)	7.4 (2.4, 12.6)	56 (18.2, 96.9)
	Ovarian cancer	-	-	-	-	-	-	3397 (908, 6081)	13.7 (3.6, 23.7)	160.8 (42.8, 287)
	Pancreatic cancer	2947 (−63, 6871)	4.1 (−0.1, 9.8)	69.6 (−1.3, 163)	1520 (−50, 3587)	3.8 (−0.1, 9.3)	76.4 (−2.3, 180)	1428 (1, 3229)	4.5 (0, 10.3)	63 (0.3, 140.5)
	Thyroid cancer	722 (503, 953)	16.7 (12.7, 20.5)	18 (12.6, 23.8)	379 (272, 500)	16.9 (12.8, 20.9)	20.1 (14.4, 26.5)	343 (233, 468)	16.5 (12.7, 20.4)	16.1 (11, 22)
	Uterine cancer	-	-	-	-	-	-	4688 (3310, 6207)	43 (31.3, 54.9)	210.8 (149.7, 278.7)
High fasting plasma glucose	All cancers	39,946 (4687, 71,669)	3.5 (0.4, 6.3)	899.8 (102.5, 1626.6)	21,566 (3550, 38,460)	3.3 (0.5, 5.9)	1024.6 (170.9, 1818.9)	18,380 (1135, 34,453)	3.8 (0.2, 7)	787.6 (40.2, 1494.8)
	Bladder cancer	1460 (−200, 3122)	6.4 (−0.9, 13.6)	30.2 (−4.2, 64.6)	1112 (−151, 2372)	6.7 (−0.9, 14.5)	50.1 (−6.9, 107.2)	348 (−49, 746)	5.5 (−0.8, 11.5)	13.2 (−1.9, 28.3)
	Breast cancer	-	-	-	-	-	-	4113 (−1265, 9443)	4.5 (−1.3, 10.3)	190.2 (−58.5, 434.2)
	Colon and rectum cancer	10,508 (5448, 15,553)	7.8 (4.1, 11.4)	235.7 (120.7, 351.1)	6323 (3244, 9494)	8.3 (4.2, 12.3)	302.4 (154.4, 452.2)	4184 (2175, 6389)	7.2 (3.7, 10.5)	175 (89.8, 268.1)
	Liver cancer	1474 (178, 2843)	2.9 (0.4, 5.4)	34 (4, 65.4)	860 (97, 1711)	2.4 (0.3, 4.6)	41.7 (4.7, 83.3)	614 (79, 1216)	4.1 (0.5, 8)	26.8 (3.4, 52.8)
	Pancreatic cancer	17,055 (2158, 30,044)	23.6 (3, 42.4)	381.2 (47.9, 672.5)	9910 (1247, 17,763)	24.8 (3.1, 44)	472.2 (59.1, 848.4)	7145 (908, 13,003)	22.2 (2.9, 39.6)	297.6 (37.6, 540.9)
	Tracheal, bronchus, and lung cancer	5336 (−1080, 11,682)	2.6 (−0.5, 5.6)	119.7 (−24.2, 260.1)	3361 (−649, 7282)	2.7 (−0.5, 5.8)	158.2 (−30.5, 342.6)	1975 (−411, 4309)	2.3 (−0.5, 5.1)	84.9 (−17.5, 185.7)
**Environmental/** **occupational risks**	All cancers	91,965 (75,474, 107,337)	8.1 (6.7, 9.2)	1991.8 (1640.6, 2322.8)	70,628 (56,824, 83,689)	10.8 (8.7, 12.7)	3219.4 (2578.3, 3813.8)	21,337 (16,155, 26,491)	4.4 (3.5, 5.3)	895.3 (684.9, 1113.5)
	Larynx cancer	843 (501, 1196)	16.5 (9.9, 22.6)	18 (10.5, 25.6)	798 (467, 1143)	18 (10.6, 25)	36.1 (20.9, 51.7)	45 (24, 72)	6.7 (3.7, 10.3)	1.9 (1, 3)
	Leukemia	287 (76, 491)	0.6 (0.2, 1.1)	9 (2.4, 15.4)	147 (42, 261)	0.5 (0.1, 0.9)	9.2 (2.6, 16.1)	139 (33, 240)	0.8 (0.2, 1.3)	8.9 (2.1, 15.3)
	Mesothelioma	15,110 (13,797, 16,250)	97.4 (96.8, 98)	328.3 (300.3, 352.6)	12,463 (11,430, 13,375)	98.1 (97.4, 98.6)	569.8 (523.4, 610.4)	2647 (2266, 2956)	94.3 (92.5, 95.7)	114.7 (99.1, 127.8)
	Ovarian cancer	-	-	-	-	-	-	2189 (1073, 3457)	8.8 (4.3, 14)	84.1 (41.6, 133)
	Tracheal, bronchus, and lung cancer	73,525 (57,369, 88,275)	35.3 (28.2, 41.2)	1591.3 (1249.3, 1919.9)	57,211 (43,816, 70,627)	46.2 (35.9, 55)	2603.8 (1978.6, 3211.8)	16,314 (11,692, 21,163)	19.3 (14.4, 24.2)	685.6 (494.8, 878.6)
* Occupational carcinogens*	All cancers	82,345 (66,310, 96,601)	7.2 (5.8, 8.4)	1763.1 (1419.1, 2069.8)	65,868 (51,880, 78,213)	10.1 (8, 12)	2980 (2336.1, 3554.8)	16,477 (11,845, 21,079)	3.4 (2.6, 4.2)	676.4 (488.2, 861.2)
	Larynx cancer	843 (501, 1196)	16.5 (9.9, 22.6)	18 (10.5, 25.6)	798 (467, 1143)	18 (10.6, 25)	36.1 (20.9, 51.7)	45 (24, 72)	6.7 (3.7, 10.3)	1.9 (1, 3)
	Leukemia	287 (76, 491)	0.6 (0.2, 1.1)	9 (2.4, 15.4)	147 (42, 261)	0.5 (0.1, 0.9)	9.2 (2.6, 16.1)	139 (33, 240)	0.8 (0.2, 1.3)	8.9 (2.1, 15.3)
	Mesothelioma	15,110 (13,797, 16,250)	97.4 (96.8, 98)	328.3 (300.3, 352.6)	12,463 (11,430, 13,375)	98.1 (97.4, 98.6)	569.8 (523.4, 610.4)	2647 (2266, 2956)	94.3 (92.5, 95.7)	114.7 (99.1, 127.8)
	Ovarian cancer	-	-	-	-	-	-	2189 (1073, 3457)	8.8 (4.3, 14)	84.1 (41.6, 133)
	Tracheal, bronchus, and lung cancer	63,904 (47,445, 76,925)	30.7 (23.9, 36.6)	1362.6 (1009.4, 1647.2)	52,450 (38,350, 64,990)	42.3 (31.9, 51.5)	2364.5 (1716, 2935.1)	11,453 (7635, 15,538)	13.5 (9.3, 18)	466.7 (313, 631.8)
* Particulate matter pollution*	Tracheal, bronchus, and lung cancer	11,613 (5756, 18,636)	5.6 (2.9, 8.8)	267.3 (133, 429)	6908 (3438, 11,011)	5.6 (2.9, 8.8)	332.1 (166.2, 530.5)	4705 (2338, 7399)	5.6 (2.9, 8.8)	209.2 (104.2, 330.2)
* Residential radon*	Tracheal, bronchus, and lung cancer	2404 (−1024, 8642)	1.2 (−0.5, 4.2)	55.4 (−23.5, 199.2)	1430 (−610, 5181)	1.2 (−0.5, 4.2)	68.8 (−29.2, 248.3)	974 (−413, 3516)	1.2 (−0.5, 4.2)	43.3 (−18.4, 156.3)

**Risk column**: Bold = GBD level-1 risks, italics = GBD level-3 risks, other GBD level-2 risks. The “All cancers” category excludes non-melanoma skin cancer. ASR, age-standardized DALY rate. Numbers in brackets represent 95% uncertainty intervals (UI). % denotes proportion of DALYs from the specific cancer attributable to the risk factor. Malignant skin melanoma, non-melanoma skin cancer, testicular cancer, brain and central nervous system cancer, Hodgkin lymphoma, hepatoblastoma, eye cancers, soft tissue and other extraosseous sarcomas, malignant neoplasm of bone and articular cartilage, and neuroblastoma and other peripheral nervous cell tumors were not estimated either due to lack of data or absence of associated risk.

## Data Availability

The data were extracted from the Global Health Exchange website (https://ghdx.healthdata.org/, accessed on 13 January 2025). Further interactive exploration of GBD2021 results on cancer burden can be done here: https://vizhub.healthdata.org/gbd-compare/cancer. The GBD data and tools guide can be accessed here: https://www.healthdata.org/research-analysis/about-gbd/gbd-data-and-tools-guide. To view, download, and use GBD results, click here: https://www.healthdata.org/data-tools-practices/interactive-visuals/gbd-results.
